# Thermoneutral housing shapes hepatic inflammation and damage in mouse models of non-alcoholic fatty liver disease

**DOI:** 10.3389/fimmu.2023.1095132

**Published:** 2023-02-17

**Authors:** Jarren R. Oates, Keisuke Sawada, Daniel A. Giles, Pablo C. Alarcon, Michelle S.M.A. Damen, Sara Szabo, Traci E. Stankiewicz, Maria E. Moreno-Fernandez, Senad Divanovic

**Affiliations:** ^1^ Department of Pediatrics, University of Cincinnati College of Medicine, Cincinnati, OH, United States; ^2^ Division of Immunobiology, Cincinnati Children's Hospital Medical Center, Cincinnati, OH, United States; ^3^ Immunology Graduate Program, Cincinnati Children’s Hospital Medical Center and the University of Cincinnati College of Medicine, Cincinnati, OH, United States; ^4^ Medical Scientist Training Program, University of Cincinnati College of Medicine, Cincinnati, OH, United States; ^5^ Division of Pathology, Cincinnati Children’s Hospital Medical Center, Cincinnati, OH, United States; ^6^ Center for Inflammation and Tolerance, Cincinnati Children’s Hospital Medical Center, Cincinnati, OH, United States

**Keywords:** temperature, MCD, WD+CCl_4_, NAFLD, NASH, immune cells, inflammation

## Abstract

**Introduction:**

Inflammation is a common unifying factor in experimental models of non-alcoholic fatty liver disease (NAFLD) progression. Recent evidence suggests that housing temperature-driven alterations in hepatic inflammation correlate with exacerbated hepatic steatosis, development of hepatic fibrosis, and hepatocellular damage in a model of high fat diet-driven NAFLD. However, the congruency of these findings across other, frequently employed, experimental mouse models of NAFLD has not been studied.

**Methods:**

Here, we examine the impact of housing temperature on steatosis, hepatocellular damage, hepatic inflammation, and fibrosis in NASH diet, methionine and choline deficient diet, and western diet + carbon tetrachloride experimental models of NAFLD in C57BL/6 mice.

**Results:**

We show that differences relevant to NAFLD pathology uncovered by thermoneutral housing include: (i) augmented NASH diet-driven hepatic immune cell accrual, exacerbated serum alanine transaminase levels and increased liver tissue damage as determined by NAFLD activity score; (ii) augmented methionine choline deficient diet-driven hepatic immune cell accrual and increased liver tissue damage as indicated by amplified hepatocellular ballooning, lobular inflammation, fibrosis and overall NAFLD activity score; and (iii) dampened western diet + carbon tetrachloride driven hepatic immune cell accrual and serum alanine aminotransferase levels but similar NAFLD activity score.

**Discussion:**

Collectively, our findings demonstrate that thermoneutral housing has broad but divergent effects on hepatic immune cell inflammation and hepatocellular damage across existing experimental NAFLD models in mice. These insights may serve as a foundation for future mechanistic interrogations focused on immune cell function in shaping NAFLD progression.

## Introduction

The unabated obesity pandemic (~1.5 billion people obese globally) is accompanied by a concomitant increase in the prevalence of non-alcoholic fatty liver disease (NAFLD). NAFLD, which affects approximately 25-30% of obese individuals, is considered the most common chronic liver disease and a leading cause for needing liver transplantation (1, 2). NAFLD encompasses a broad spectrum of liver conditions ranging from steatosis to non-alcoholic steatohepatitis (NASH) to cirrhosis, which eventually can progress to hepatocellular carcinoma (HCC) (1, 3). To study the broad aspects of NAFLD progression the field has traditionally employed a variety of experimental animal models. Notably, in mouse models of NAFLD, the strengths of each model are focused on select key parameters represented in human disease (e.g., steatosis, steatohepatitis, hepatocyte ballooning, Mallory-Denk bodies and fibrosis).

Prominent mouse models of NAFLD involve various dietary challenges including high fat diet (HFD), methionine choline deficient (MCD) diet, and chemical perturbations in combination with western diet (WD) (e.g., Carbon tetrachloride [CCl_4_] + WD diet) (3–6). HFD feeding drives robust obesity and hepatic recruitment of Kupffer cells and neutrophils, and only causes mild inflammation and minimal fibrosis (the latter only evident after prolonged HFD feeding) (7, 8). Conversely, MCD diet consistently induces robust hepatic immune cell recruitment, liver damage, and development of hepatic fibrosis, without the induction of obesity (9–11). Chemical perturbations, including high-dose CCl_4_, promote hepatic inflammation and fibrosis with induction of hepatocellular necrosis that is not consistent with human liver disease pathogenesis (9). As such, CCl_4_ is used at lower doses and in combination with WD feeding to recapitulate histopathological manifestations of human NAFLD more accurately. However, unlike in human disease, this model is associated with weight loss (5). Nevertheless, despite being traditionally employed in studying NAFLD pathogenesis, the above discussed models only partly recapitulate clinical human disease progression. Notably, these shortcomings may limit the discovery of key cellular and molecular mechanisms underlying liver disease pathogenesis. Given that inflammation represents a unifying component of NAFLD progression (11–14) across the models used, further improvement of existing experimental models to enable discovery of mechanisms that shape inflammatory responses in NAFLD may represent the critical locus of the effect for translation of key discoveries to clinical relevance.

Ambient housing temperature regulates inflammatory responses to internal and external stimuli (15, 16). Common animal facilities house mice in thermo-stress (Ts) (20-23˚C) conditions. Such conditions however are associated with the activation of cold stress responses in mice, augmented production of stress hormone (e.g., corticosterone and catecholamine) which drives a multitude of physiological and metabolic changes (17, 18). Importantly, Ts housing suppresses immune responses including altered cellular energy availability, cytokine production, and lymphocyte egress from lymph nodes (15, 19–21). Conversely, thermoneutral (Tn) housing (29-34˚C), a temperature where mice do not need to expend excess energy to maintain core body temperature, reverses the suppression of immune responsiveness observed at Ts housing conditions (1, 3, 16, 22). In the context of NAFLD, Tn housing coupled with HFD feeding accelerates and exacerbates disease progression and pathogenesis. Specifically, Tn housing in combination with HFD feeding enhances hepatic steatosis and inflammation, and hepatocellular damage in wild type C57BL/6 male mice and hepatic fibrosis in AKR mice (3). Further, wild type C57BL/6 female mice, which are generally resistant to HFD-induced obesity when housed at Ts conditions, developed robust obesity and NAFLD when housed at Tn conditions (3). Thus, the ability of Tn housing to reverse paradigms seen at Ts conditions by restricting immune suppression and promoting obesity in female mice warrants the examination of the impact of Tn housing on hepatic inflammation, steatosis, liver damage and development of hepatic fibrosis across multiple, traditionally used, experimental models of NAFLD in mice.

Here, we show that Tn housing shapes hepatic inflammation across multiple, traditionally used, experimental mouse models of NAFLD. In a model utilizing NASH diet, Tn housing augments obesity, serum ALT, hepatic immune cell accrual, expression of immune cell recruiting chemokines which correlates with increased lobular inflammation and liver disease as determined by NAFLD Activity Score (NAS). Similarly, in the MCD diet driven NAFLD model, Tn housing promotes increased hepatic expression of immune cell recruiting chemokines correlating with hepatic immune cell accrual, increased expression of fibrosis-associated genes and worsened fibrosis as indicated by increased cholangiolar proliferations accompanied by thicker collagenous pericellular fibers and overall increased NAS severity. Lastly, in the context of chemical perturbations combined with WD diet induced obesity, Tn housing decreases serum ALT which correlates with a decrease in hepatic immune cell accrual and modified immune cell inflammatory capacity. Collectively, these findings demonstrate for the very first time that thermoneutral housing has broad, yet divergent effects on hepatic immune cell inflammation and hepatocellular damage across existing experimental NAFLD models in C57BL/6 mice. As studies utilizing thermoneutral housing become more prevalent, insights gained may serve as a foundation for interrogation of mechanisms instructing immune cell function and development of future therapies to NAFLD.

## Materials and methods

### Mice and dietary studies

Wild type (WT) mouse breeding pairs, originally purchased from Jackson Laboratories, were on C57BL/6J background, housed at thermo-stress (Ts; 22°C) conditions with free access to autoclaved food and water, and bred at Cincinnati Children’s Hospital Medical Center (CCHMC) in a specific pathogen-free (spf) facility. Only 8-week-old WT male mice were used in our studies. For our studies, 6-week old mice were maintained at Ts or placed at thermoneutral (Tn; 30°C with 30% humidity) conditions for 2 weeks to allow acclimation prior to the initiation of dietary challenge. Tn housing was accomplished using Caron chambers (Caron Products & Services, INC). For all studies, food and water were replaced weekly. Body weight was recorded weekly. Corn cob bedding was used in the housing of all mice. All animal care was provided in accordance with the Guide for the Care and Use of Laboratory Animals. All studies were approved by the Cincinnati Children’s Hospital Medical Center IACUC.

#### Chow diet

WT C57BL/6 mice (8-week-old) were fed CD (fat 13.5% kcal, carbohydrate 59% kcal, protein 27.5% kcal; LabDiet 5010) and housed at either Ts or Tn as controls to specific dietary challenge experiments.

#### Methionine-choline deficient diet

WT C57BL/6 mice (8-week-old) were fed MCD diet (MCD; Research Diets #A02082002B; 16% Protein, 63% Carbohydrate and 21% Fat kcal/gram) and housed at either Ts or Tn for 4 or 10 weeks as previously described (10, 23).

#### NASH diet

WT C57BL/6 mice (8-week-old) were fed a NASH diet (40% kcal fat, 20% kcal fructose, and 2% kcal cholesterol by weight; Research diets, D09100301) and housed at either Ts or Tn for 22 weeks as previously described (24).

#### Carbon tetrachloride (CCl_4_)

WT C57BL/6 mice (8-week-old) were fed CD. CCl_4_ (Sigma-Aldrich, 289116-100ML) was injected with the dose of 2 µl (0.32 µg)/g of body weight, of CCl_4_ or olive oil (control) i.p. 2x weekly for 3 weeks.

#### Western diet + carbon tetrachloride (WD+CCl_4_)

WT C57BL/6 mice (8-week-old) were fed a WD (21.1% fat, 41% Sucrose, and 1.25% Cholesterol by weight; Teklad diets, TD. 120528) and a high sugar solution (23.1 g/L d-fructose and 18.9 g/L d-glucose) in drinking water. CCl_4_ (Sigma-Aldrich, 289116-100ML), 0.2 µl (0.32 µg)/g of body weight or corn oil (control) was i.p. injected once per week, as previously described (5, 24) for 12 weeks.

### Hepatic immune cell isolation

Hepatic immune cells were isolated using Miltenyi Biotec Gentlemax technology followed by Percoll gradient. Briefly, whole liver was homogenized using Miltenyi Gentlemax C tubes using RPMI + 10% fetal bovine serum. After resuspension, cells were centrifuged at 2000 rpm for 10 minutes. Cell pellets were homogenized in a 33% Percoll solution (Sigma-Aldrich) diluted in RPMI medium 1640 (Gibco). Following gradient separation, and lysing of red blood cells, hepatic infiltrating immune cells were subsequently analyzed by flow cytometry (3, 25, 26).

### Hepatic function

Hepatic triglycerides (TGs) were quantified using Triglyceride Reagent and Triglyceride Standards (Pointe Scientific). Serum alanine aminotransferase (ALT) and aspartate aminotransferase levels (AST) were quantified using ALT Reagent, AST Reagent and Catatrol I and II (Catachem). For histology, liver tissue was fixed in 10% buffered formalin, and stained with H&E or Masson’s trichrome and evaluated by a board-certified pathologist (3, 24).

### Hepatic immune cell characterization

To determine immune cell population single cell suspensions derived from hepatic tissues, isolated immune cells were labeled with monoclonal antibodies. For cytokine production, total single cells were stimulated for 4 hours with Phorbol 12-myristate 13-acetate (PMA; 50 ng/ml) (Sigma-Aldrich, St. Louis, MO) and Ionomycin (1 μg/ml) (EMD Millipore), in presence of Brefeldin A (10 μg/mL) (GoldBio). Subsequently, data were collected using an LSR Fortessa (BD) and Cytek Aurora (Cytek Biosciences) and analyzed using FlowJo X software (vX10).

### Immune cell characterization was as follows

#### CD4^+^ T cells

Mouse cells were stained with Live/Dead stain (Zombie UV Dye: Biolegend) and with directly-conjugated monoclonal antibodies to CD45-PE-Dazzle594 (Biolegend, 104), TCRβ-APCef780 or APC (Invitrogen, H57-597), CD4-APC-ef780 or BV786 (e-Biosciences, RM4-5), then fixed, permeabilized and stained for the cytokines IL-17A-PerCpCy5.5 or PE (e-Biosciences, 17B7), IFNγ-PE-Cy7 (e-Biosciences, XMG1.2) and TNFα-BV650 (Biolegend, MP6-XT22).

#### CD8^+^ T cells

Mouse cells were stained with Live/Dead stain (Zombie UV Dye: Biolegend) and with directly-conjugated monoclonal antibodies to CD45-PE-Dazzle594 (Biolegend, 104), TCRβ-APCef780 or APC (Invitrogen, H57-597), CD8-BV510 (Biolegend, 53-6.7), then fixed, permeabilized and stained for the cytokines IL-17A-PerCpCy5.5 or PE (e-Biosciences, 17B7), IFNγ-PE-Cy7 (e-Biosciences, XMG1.2) and TNFα-BV650 (Biolegend, MP6-XT22).

#### Macrophages

Mouse cells were stained with Live/Dead stain (Zombie UV Dye: Biolegend) and with directly conjugated monoclonal antibodies to CD45-PE-Dazzle594 (Biolegend, 104), CD11b-eF450 (Biolegend, 17A2), F4/80-APC (eBiosciences, BM8) then fixed, permeabilized and stained for the cytokine TNFα-BV650 (Biolegend, MP6-XT22).

#### NK cells

Mouse cells were stained with Live/Dead stain (Zombie UV Dye: Biolegend) and with directly conjugated monoclonal antibodies to CD45-PE-Dazzle594 (Biolegend, 104), NK1.1-BV711 (Biolegend, PK136) then fixed, permeabilized and stained for the cytokine IFNγ-PE-Cy7 (e-Biosciences, XMG1.2).

#### NKT cells

Mouse cells were stained with Live/Dead stain (Zombie UV Dye: Biolegend) and with directly conjugated monoclonal antibodies to CD45-PE-Dazzle594 (Biolegend, 104), NK1.1-BV711 (Biolegend, PK136) and TCRβ-APCef780 or APC (Invitrogen, H57-597).

#### B cells

Mouse cells were stained with Live/Dead stain (Zombie UV Dye: Biolegend) and with directly conjugated monoclonal antibodies to CD45-PE-Dazzle594 (Biolegend, 104) and B220-BV605 (Biolegend, RA3-6B2).

#### Neutrophils

Mouse cells were stained with Live/Dead stain (Zombie UV Dye: Biolegend) and with directly conjugated monoclonal antibodies to CD45-PE-Dazzle594 (Biolegend, 104), CD11b-eF450 (Biolegend, 17A2), Gr1-FITC (Invitrogen, RB6-8C5) then fixed, permeabilized and stained for the cytokine TNFα-BV650 (Biolegend, MP6-XT22).

**Table:** 

Primer	Forward Sequence 5’-3’	Reverse Sequence 5’-3’
Ccl2	AGATGCAGTTAACGCCCCAC	TGTCTGGACCCATTCCTTCTTG
Ccl3	ACCATGACACTCTGCAACCAAG	TTGGAGTCAGCGCAGATCTG
Tnfa	CCAGACCCTCACACTCAGATCA	CACTTGGTGGTTTGCTACGAC
Ifng	TGGCTGTTTCTGGCTGTTACTG	ACGCTTATGTTGTTGCTGATGG
Il6	TGGTACTCCAGAAGACCAGAGG	AACGATGATGCACTTGCAGA
Ki67	ATCATTGACCGCTCCTTTAGGT	GCTCGCCTTGATGGTTCCT
Ccnd1	GCGTACCCTGACACCAATCTC	CTCCTCTTCGCACTTCTGCTC
Col1a1	GGTTTCCACGTCTCACCATT	ACATGTTCAGCTTTGTGGACC
Col1a2	AGCAGGTCCTTGGAAACCTT	AAGGAGTTTCATCTGGCCCT
Acta2	GTTCAGTGGTGCCTCTGTCA	GGGATCCTGACGCTGAAGTA
H19	CAACATCCCACCCACCGTAA	GCTCACCAAGAAGGCTGGAT
Afp	TCTTTCCACTCCACTTTGGC	GGCGATGGGTGTTTAGAAAG
Chrebp	TCTGCAGATCGCGTGGAG	CTTGTCCCGGCATAGCAAC
Srebp	CTGTCTCACCCCCAGCATAG	GATGTGCGAACTGGACACAG
Lxra	CTCAATGCCTGATGTTTCTCCT	TCCAACCCTATCCCTAAAGCAA
Lipe	TCTCGTTGCGTTTGTAGTGC	ACGCTACACAAAGGCTGCTT
Pparg	GAATGCGAGTGGTCTTCCAT	TGCACTGCCTATGAGCACTT
Irs1	CTCCTGCTAACATCCACCTTG	AGCTCGCTAACTGAGATAGTCAT
B actin	GGCCCAGAGCAAGAGAGGTA	GGTTGGCCTTAGGTTTCAGG

### mRNA and qPCR analysis

Tissue samples were homogenized in TRIzol. RNA was extracted, reverse transcribed to complementary DNA (cDNA), and subjected to qPCR analysis (Light Cycler 480 II; Roche Diagnostics) as previously described (3, 15). The primer sequences, all from Invitrogen are as follows see below:

### Statistical analysis

For statistical analysis, normality and lognormality tests and parametric tests were employed as determined by the Graphpad Prism software. A 2-tailed student’s *t* test was used when the comparison was between 2 groups, while a 1-way ANOVA with Tukey’s *post hoc* test to assess differences between specific groups was employed for 3 or more groups. Statistical analysis was completed using Prism 5a (GraphPad Software Inc.). All values are represented as means ± SEM. A *P*-value less than 0.05 was considered significant.

## Results

### Tn housing augments NASH diet driven myeloid immune cell accrual in the liver and exacerbates liver disease pathogenesis

**Figure 1:**
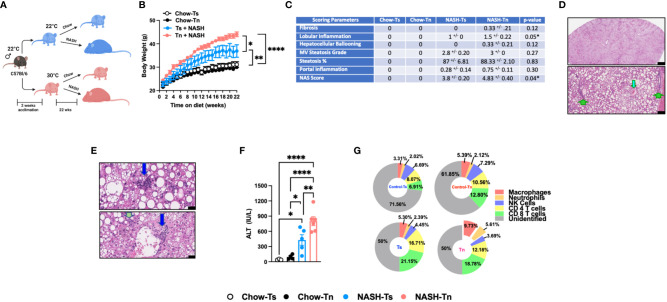
Tn housing augments NASH diet driven myeloid immune cell accrual in the liver and exacerbates liver disease pathogenesis. **(A)** Schematic of the experimental design. Eight-week-old WT mice maintained at Ts or acclimated to Tn for 2 weeks prior to study initiation were fed a chow (baseline reference) or NASH diet for 22 weeks. During the course of 22 weeks, **(B)** body weights of Ts and Tn housed mice were recorded. At the conclusion of the study, additional parameters of NAFLD severity were analyzed. **(C)** The liver tissue was preserved in formalin, stained with hematoxylin and eosin (H&E), and analyzed by a clinical pathologist. Table depicting histological scoring analyses for fibrosis, lobular inflammation, hepatocellular ballooning, macrovesicular (MV) steatosis grade, steatosis percentage, portal inflammation and NAFLD activity score (NAS) severity. **(D)** (Top) Extensive steatosis (80-95%) on H&E with a combination of classic large droplet macrovesicular steatosis (LD-MS) and small droplet (SD-MS) macrovesicular steatosis. (Portae: green arrows. Central veins: light blue arrows) (Top) Black bar = 629µm; (Bottom) Black bar = 109µm. **(E)** Liver H&E staining. (Top) Ballooning (yellow arrows), in a mixed patterned background with overlapping features, including SD-MS (light blue arrows) and LD-MS. Black bar = 25µm (Middle) Example for focal lobular inflammation (lymphocytic) (blue arrows), classic for NAFLD. Black bar = 25µm. (Bottom) Focal portal inflammation, perivenular (blue arrow), with small focus of cholangiolar reactivity (green arrow). Black bar = 33µm. **(F)** Hepatocellular damage as quantified by serum ALT levels collected at the conclusion of the study. **(G)** Hepatic inflammation as defined by flow cytometric analyses of hepatic immune cell accrual at the conclusion of the study. Donut charts depicting liver immune compositions (macrophages, neutrophils, CD4^+^ cells, CD8^+^ cells, NK cells, and unidentified cells) as determined by flow cytometry from percent of CD45^+^ population. In bar graphs, data represent mean +/- SEM. **(B, C, F, G)** Representative of 2 individual experiments (n=5-6/condition). **(F)** One-way ANOVA. *P<0.05, ** P<0.01, **** P<0.0001.

Our initial investigation focused on determining the impact of Tn housing on liver damage at homeostatic conditions. Tn housing did not impact body weight and promoted a slight reduction in wet liver weights, compared to Ts. Importantly, both Tn and Ts housed animals displayed similar serum ALT levels (**Supplementary Figures 1A–C**). As coupling high fat diet (HFD)-driven obesity with Tn housing exacerbates inflammatory responsiveness and accelerates NAFLD pathogenesis (3), we next examined the impact of Tn housing in a model of obesogenic diet-driven NAFLD, a diet rich in cholesterol and sugars that has been postulated to mimic human disease (27–29). To mimic these conditions, Ts and Tn housed C57BL/6 wild type male mice were fed a NASH diet for 22 weeks (**Figure 1A**). Tn housed mice, compared to Ts housed counterparts, had increased total body weight gain over time (p=0.01) (**Figure 1B**). Further examination of visceral white adipose tissue (WAT) depots revealed that Tn housing dominantly impacted perirenal WAT fat redistribution (p=0.04) rather than inguinal and epididymal WAT depots (**Supplementary Figure 2A**).

Given the observed changes in obesity and adiposity in Tn housed mice, we analyzed the impact of Tn housing on gross liver histology. Obesity is associated with ectopic fat deposition in the liver, thus the impact of Tn housing on NASH diet-driven hepatic steatosis and hepatocellular damage was examined. Both Tn and Ts housed, NASH diet fed mice displayed equal presence of classic large droplet macrovesicular steatosis (LD-MS; a single large steatotic droplet replaces nearly the entire cytoplasm and pushes the nucleus peripherally) and small droplet macrovesicular steatosis (SD-MS; numerous smaller droplets of fat that replace essentially the entire cytoplasm and expand the cell) (**Figures 1C, D**) (see arrows). Congruently, both Tn and Ts housed, NASH diet fed mice had similar wet liver weights (**Supplementary Figure 2B**) and hepatic triglyceride accumulation (**Supplementary Figure 2C**). However, additional histological examination revealed that despite similar hepatocyte ballooning, Tn housed NASH-diet-fed mice, compared to Ts housed counterparts, exhibited increased focal lobular inflammation (p=0.05), and had an overall increase in NAFLD Activity Score (NAS) severity (p=0.04) (**Figures 1C**, depicted in E) (see arrows). Lastly, in agreement with histological analyses, the Tn housed, NASH diet fed mice, compared to Ts housed counterparts, had exacerbated serum alanine transaminase (ALT) levels (p=0.01) (**Figure 1F**).

Hepatocellular damage is closely linked with altered hepatic inflammation. Hence, we next examined the impact of Tn housing on NASH diet-driven hepatic inflammation, hepatic immune cell composition and immune cell inflammatory capacity. Tn and Ts housed, NASH diet fed mice had similar hepatic mRNA expression of *Tnfa*, a proinflammatory cytokine that contributes to hepatic inflammation and damage (30, 31) (**Supplementary Figure 2D**). However, Tn housing increased hepatic mRNA expression of immune cell recruiting chemokines *Ccl2* (p=0.000009) and *Ccl3* (p=0.04) (**Supplementary Figure 2E**). Subsequent analysis of the immune cell composition demonstrated that Tn housing promoted an increase in total hepatic immune cell accrual (p=0.04) in context of NASH diet feeding (**Supplementary Figure 2F**). Cellular characterization of hepatic immune infiltrate in Tn housed NASH-diet-fed mice, compared to Ts housed counterparts, revealed specific increase in frequencies and absolute numbers of macrophages (CD45^+^CD11b^+^F4/80^+^) (p=0.03) and neutrophils (CD45^+^CD11b^+^Gr1^+^) (p=0.03), but reduced frequency of CD4^+^ T cells (CD45^+^CD3^+^CD4^+^) (**Figure 1G** and **Supplementary Table 1**, for gating strategy see **Supplementary Figure 3**). However, despite the apparent changes in hepatic immune cell composition, the inflammatory capacity of hepatic immune cells following PMA/ionomycin stimulation (**Supplementary Figures 2G, H**) was similar in Tn and Ts housed, NASH diet fed mice.

Prolonged hepatic inflammation induces tissue damage and development of hepatic fibrosis (32, 33). However, traditional HFD feeding does not induce robust hepatic fibrosis in C57BL/6 wild type mice under Ts housing conditions. Notably, diets high in cholesterol and sugar (i.e., fructose and sucrose) content are known to yield upregulation of pathways associated with fibrosis and promote histological patterns of fibrosis that mimic fibrosis-like characteristics seen in human disease (34, 35). Hence, the impact of Tn housing on hepatic fibrosis was examined. Tn and Ts housed NASH-diet-fed mice had similar expression of fibrosis associated genes *Col1a1*, *Col1a2* and *Acta2* in the liver (**Supplementary Figure 2I**). Congruently, both Tn and Ts housed, NASH diet fed mice had minimal fibrosis in association with periportal cholangiolar reactivity as determined by trichrome staining (**Figure 1C** and **Supplementary Figure 2J**).

NAFLD is also associated with dysregulated lipid metabolism and insulin signaling, both of which contribute to development and severity of metabolic syndrome (36, 37). Obesity in particular is associated with dysregulation of lipid metabolism and insulin signaling pathways (36, 37). Hence, the impact of Tn housing on pathways associated with lipid metabolism and insulin signaling were next examined. Notably, Tn and Ts housed, NASH diet fed mice, despite differences in NAFLD severity, had similar hepatic expression of insulin signaling (*Pparγ* and *Irs1*) and lipid metabolism-associated genes (*Lxrα*, *Lipe*, *Chrebp*, and *Srebp*) (**Supplementary Figures 2K–L**).

### Tn housing augments MCD diet driven myeloid immune cell accrual in the liver and exacerbates liver disease pathogenesis

**Figure 2:**
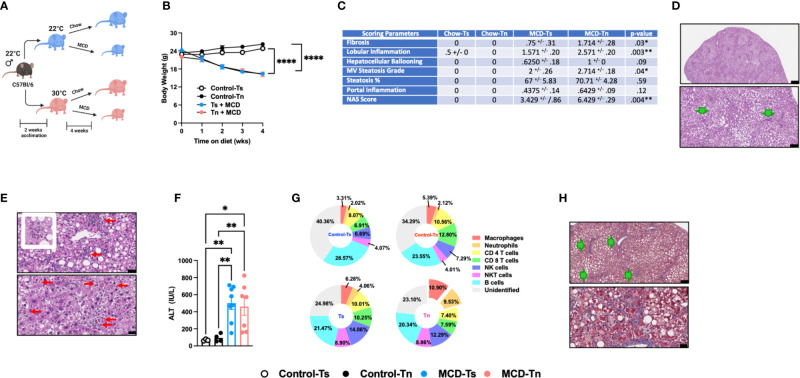
Tn housing augments MCD diet driven hepatic myeloid immune cell accrual in the liver and exacerbates liver disease pathogenesis. **(A)** Schematic of the experimental design. Eight-week-old WT mice maintained at Ts or acclimated to Tn for 2 weeks and fed a chow (baseline reference) or MCD diet for 4 weeks. During the course of the 22 weeks, **(B)** body weights of Ts and Tn housed mice were recorded. **(C)** The liver tissue was preserved in formalin, stained with hematoxylin eosin (H&E), and analyzed by a clinical pathologist. Table depicting histological scoring analyses for fibrosis, lobular inflammation, hepatocellular ballooning, macrovesicular (MV) steatosis grade, steatosis percentage, portal inflammation and NAFLD activity score (NAS) severity. **(D)** Liver H&E staining. At low power, zone 2-predominant macrovesicular steatosis is noted diffusely (top and bottom) (For architectural orientation, green arrows point to portae.) Top, black bar = 332µm; Bottom, black bar = 119µm. **(E)** Liver hematoxylin eosin (H&E) staining. (Top) Reactive cholangiolar proliferations, with inflammation, some microaggregating (red arrows) and occasionally “microgranulomatous” (insert). Black bar = 34µm. (Bottom) Partial pericentral/zone 3-sparing, though with spotty hepatocellular necrosis (red arrows) that are occasionally clustering. Black bar = 25µm. **(F)** Hepatocellular damage as quantified by serum ALT levels collected at the conclusion of the study. **(G)** Hepatic inflammation as defined by flow cytometric analyses of hepatic immune cell accrual. Donut charts depicting liver immune compositions (macrophages, neutrophils, CD4^+^ cells, CD8^+^ cells, and unidentified cells as determined by flow cytometry from percent of CD45^+^ population. **(H)** Liver tissues were preserved in formalin, sectioned and trichrome stained for analysis by pathologist. (Top) Patterns of fibrosis in trichrome stain. Black bar = 119µm. (Bottom) Focally accentuated collagenous pericellular fibrosis in a periportal location. Black bar = 61µm. In bar graphs, data represent mean +/- SEM. **(B)** Data combined from 2 individual experiments (n=6-8/condition). **(C)** Data combined from 2 individual experiments, (n=8/condition). **(F, G)** Data combined from 2 individual experiments (n=8/condition). **(C)** One-way ANOVA. *P<0.05, **P<0.01, ****P<0.0001..

Increased incidence of NAFLD is reported to also occur in the absence of obesity (38). Using the MCD diet model of NAFLD, which induces weight loss but promotes hepatic steatosis, inflammation, and hepatocellular damage, we next examined whether Tn housing enhanced NAFLD pathogenesis in an obesity independent setting. Tn and Ts housed C57BL/6 wild type male mice were fed MCD diet for 4 weeks (**Figure 2A**). Tn housing did not impact MCD diet driven weight loss – a key characteristic of MCD diet feeding (**Figure 2B**). Hepatic triglycerides levels were similar despite increased liver to body weight ratio (p=0.005) in Tn housed, MCD diet fed mice (**Supplementary Figures 4A, B**). However, histopathological analyses revealed that Tn housed, MCD diet fed mice, compared to Ts housed counterparts, exhibited a higher macrovesicular steatosis grade (p=0.04) (**Figures 2C, D**, see green arrows). In addition, Tn housed, MCD diet fed mice, compared to Ts housed counterparts, had increased overall hepatocellular damage as evidenced by increased focal inflammation (p=0.003) and necrosis (acidophil bodies) (**Figure 2E**, see red arrows), and a significant increase in NAS severity (p=0.004) (**Figure 2C**), but had similar ALT and Aspartate aminotransferase (AST) levels (**Figure 2F** and **Supplementary Figure 4C**). Tn housed, MCD diet fed mice, compared to Ts counterparts, exhibited comparable hepatic expression levels of *Tnfa*, *Ccl2 and Ccl3* in total liver tissue (**Supplementary Figures 4D, E**). Similarly, analysis of the immune cell composition showed comparable numbers of total hepatic immune cells (**Supplementary Figure 4F**). Characterization of hepatic immune cell infiltrate revealed that Tn housing in combination with MCD diet feeding promoted a specific increase in frequencies and absolute numbers of macrophages (frequency p=0.01; absolute # p=0.01) and neutrophils (frequency p=0.0002; absolute # p=0.001) and a decrease in CD4^+^ and CD8^+^ T cells absolute numbers (**Figure 2G** and **Supplementary Table 2**). Nevertheless, despite the changes in hepatic immune cell composition, the inflammatory capacity of these cells following *ex vivo* PMA/ionomycin stimulation was not altered (**Supplementary Figures 4G, H**).

MCD diet is a well-accepted experimental model of hepatic fibrosis (9, 39, 40). Whether Tn housing modifies development of hepatic fibrosis in the context of MCD feeding is unknown. Tn and Ts housed, MCD diet fed mice exhibited similar hepatic expression of fibrosis associated genes *Col1a1*, *Col1a2* and *Acta2* (p=0.18) (**Supplementary Figure 4I**). Nevertheless, Tn housed, MCD diet fed mice, compared to Ts housed counterparts, had worsened hepatic fibrosis as indicated by increased cholangiolar proliferations accompanied by thicker collagenous pericellular fibers (**Figures 2C, H**, see green arrows). MCD diet does not induce metabolic syndrome (9, 41). We next examined whether Tn housing would be sufficient to alter the expression of lipid metabolism- and insulin signaling-associated genes linked with metabolic syndrome in NAFLD (36, 37). Notably, Tn and Ts housed, MCD diet fed mice, despite differences in NAFLD severity, exhibited similar hepatic expression of insulin signaling (*Pparγ* and *Irs1*) and lipid metabolism-associated genes (*Lxrα*, *Lipe*, *Chrebp*, and *Srebp*) (**Supplementary Figures 4J, K**).

Prolonged MCD feeding exacerbates NASH pathology (10). To further define the impact of Tn housing on prolonged MCD-driven NAFLD, C57BL/6 wild type mice were housed at Tn and Ts and fed MCD diet for 10 weeks (**Supplementary Figure 5A**). Similar to 4 weeks of MCD diet feeding, no differences in weight loss over time between Tn and Ts housed mice were observed (**Supplementary Figure 5B**). Tn housed, MCD diet fed mice, compared to Ts counterparts, had decreased liver to body weight ratio (p=0.003) and similar total hepatic triglyceride accumulation compared to Ts housed counterparts (**Supplementary Figures 5C, D**), but displayed exacerbated steatosis percentage (p=0.03) (detected in 85-95% range) (**Supplementary Figure 5E**). Additional histological examination revealed that Tn housed, MCD diet fed mice, compared to Ts housed counterparts, had increased hepatocellular ballooning (p=0.01), multifocal lobular inflammation (p=0.01), and the overall NAS severity (p=0.005) (**Supplementary Figure 5E**), yet ALT and AST remained similar (**Supplementary Figures 5F, G**). Further, extended MCD diet feeding in Tn housed mice resulted in significantly increased hepatic expression of *Tnfa* (p=0.004), *Ccl2* (p=0.02) and *Ccl3* (p=0.01) (**Supplementary Figures 5H–I**). Despite similar absolute numbers of total hepatic immune cells (**Supplementary Figure 5J**), Tn housed, MCD diet fed mice, compared to Ts housed counterparts, had a significant increase in hepatic frequency of macrophage (p=0.001) and neutrophil (p=0.01) accrual (**Supplementary Figure 5K**). Surprisingly, hepatic macrophages isolated from these mice had uniquely blunted inflammatory capacity (p=0.003) following *ex vivo* PMA/ionomycin stimulation, a finding not seen in CD4^+^ T cells (**Supplementary Figures 5L, M**). Lastly, although prolonged MCD diet feeding of mice housed at Tn increased the hepatic expression of *Col1a1* (p=0.02) and *Col1a2* (p=0.01) (**Supplementary Figure 4N**), such induction did not yield robust histological differences in hepatic fibrosis at the conclusion of the study (**Supplementary Figure 5E**).

### Tn housing restricts WD+CCl_4_ driven liver disease pathogenesis

Carbon tetrachloride (CCl_4_) is a prominent hepatotoxin known to drive robust hepatic inflammation, damage, and fibrosis (9, 40, 42, 43). Thus, we next examined how Tn housing shapes CCl_4_ treatment-driven liver damage. C57BL/6 wild type mice were housed at Tn andTs and treated with CCl_4_ i.p. 2 times a week for 3 weeks (**Supplementary Figure 6A**). Both Tn and Ts housed mice treated with CCl_4_ had similar body weights at the conclusion of the study (**Supplementary Figure 6B**) and hepatic immune cell accrual that correlated with extensive hepatocellular necrosis (**Supplementary Figures 6C, D**, see yellow arrows). However, despite similar disease pathology, Tn housed mice treated with CCl_4_, compared to Ts housed counterparts, had reduced ALT levels (p=0.001) (**Supplementary Figure 6E**). In addition, CCl_4_ treatment induced comparable hepatic *Tnfa* and *Ccl2* and *Ccl3* expression in both Tn and Ts housed mice (**Supplementary Figures 6F, G**), similar hepatic immune cell accrual (**Supplementary Figures 6H, I**), and hepatic immune cell inflammatory capacity following *ex vivo* stimulation with PMA/ionomycin (**Supplementary Figures 6J, K**). CCl_4_ is also a potent driver of hepatic fibrosis. Analysis of the whole liver tissue showed similar expression of fibrosis associated genes (*Col1a1*, *Col1a2* and *Acta2*) (**Supplementary Figure 6L**) in Tn and Ts housed mice treated with CCl_4,_ which also correlated with similar induction of sinusoidal fibrosis as determined by histopathological analyses (**Supplementary Figure 6M**).

**Figure 3:**
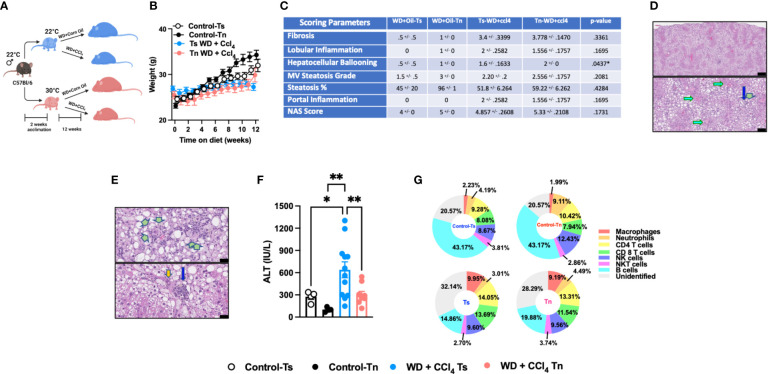
Tn housing restricts WD + CCl_4_ driven liver disease pathogenesis. **(A)** Schematic of eight-week-old WT mice maintained at Ts or acclimated to Tn for 2 weeks and treated with either WD + Corn Oil (control) or WD+CCL_4_ 2µl (0.32 µg)/g of body weight i.p. once weekly for 12 weeks. During the course of the 12 weeks, **(B)** body weight was recorded. At the conclusion of the study, additional parameters of NAFLD severity were analyzed. **(C)** The liver tissue was preserved in formalin, stained with hematoxylin eosin (H&E), and analyzed by a clinical pathologist. Table depicting histological scoring analyses for fibrosis, lobular inflammation, hepatocellular ballooning, macrovesicular (MV) steatosis grade, steatosis percentage, portal inflammation and NAFLD activity score (NAS) severity. **(D)** Liver H&E staining. (Top and bottom) Predominant distribution of large- and small-droplet macrovesicular steatosis is in zone 1 (to 2), grade 2 (to 3) range, apparent at low power. Black bar = 336µm (green arrows: portal areas and cholangiolar proliferations; light blue arrows: central veins). Black bar = 115µm. **(E)** Liver H&E staining. (Top) Cholangiolar proliferations. Periportal multilayered and solid patches (double green arrows), focal multilayered pericellular (triple arrows). Black bar = 336µm. (Bottom) Extension to lobules (single arrows), focally zone 3 (near central vein), with associated large inflammatory focus in porta (blue arrow); nuclear atypia (orange arrow). Black bar = 38µm. **(F)** Serum ALT levels at the conclusion of the study. **(G)** Hepatic inflammation as defined by flow cytometric analyses of hepatic immune cell accrual. Donut charts depicting liver immune compositions (macrophages, neutrophils, CD4^+^ cells, CD8^+^ cells, NK cells, NKT cells, B cells, and unidentified cells as determined by flow cytometry from percent of CD45^+^ population. In bar graphs, data represent mean +/- SEM. **(B, C, F, G)** Data combined from 2 individual experiments (n=10-14/condition). **(C, F)** One way ANOVA. *P<.05, **P<.01.

Western Diet (WD) feeding coupled with low dose CCl_4_ administration (WD+CCl_4_) more accurately recapitulates the histopathological manifestations of NASH with progression to cirrhosis (5). Hence, C57BL/6 wild type mice housed at Tn and Ts conditions were fed a WD coupled with low dose of CCl_4_ administration for 12 weeks (**Figure 3A**). Tn housing did not impact total body weight gain over time (**Figure 3B**), wet liver weights or hepatic triglyceride accumulation (**Supplementary Figures 7A, B**). Congruently, Tn housed mice receiving WD+CCl_4_, compared to Ts housed counterparts, had similar distribution of small droplet macrovesicular steatosis and large droplet macrovesicular steatosis (**Figures 3C, D**, see arrows). Additional histopathological analyses revealed that Tn housed, WD+CCl_4_ treated mice had increased hepatocellular ballooning (p=0.04), but similar distribution of inflammatory foci and similar overall NAS severity (**Figures 3C, E**, see green arrows). Similarly, to CCl_4_ treatment alone, Tn housed, WD + CCl_4_ treated mice exhibited decreased serum ALT (p=0.01) but not AST levels (**Figure 3F** and **Supplementary Figure 7C**). Tn housed, WD+CCl_4_ treated mice, compared to Ts housed counterparts, also had increased hepatic *Tnfa*, (p=0.03) *Ccl2* (p=0.007) and *Ccl3* (p=0.02) expression (**Supplementary Figures 7D, E**). Subsequent analyses of the immune cell composition revealed Tn housing coupled to WD+CCl_4_ treatment decreased absolute number of hepatic immune cells (p=0.05) (**Supplementary Figure 7F**), most prominently in the macrophage compartment (p=0.03) (**Figure 3G** and **Supplementary Table 3**). Nevertheless, Tn housing did not impact macrophage inflammatory capacity but rather modified the inflammatory capacity of CD4^+^ T cells following *ex vivo* stimulation with PMA/ionomycin (p=0.001) (**Supplementary Figures 7G, H**). We next examined if Tn housing modified WD + CCl_4_ treatment driven hepatic fibrosis. Increased hepatic expression of fibrosis (*Col1a1*, (p=0.31) *Col1a2* (p=0.51)*, Acta2* (p=0.05)), cell regeneration (*Ccnd1* (p=0.002) and *Ki67* (p=0.0005)) and HCC-associated genes (*Afp* (p=0.009) and *H19* (p=0.12)) was observed in Tn housed, WD + CCl_4_ treated mice, compared to Ts housed counterparts (**Supplementary Figures 7I, K**). Nonetheless, robust histological differences in pericellular delicate fibrosis (see green arrows) or HCC development as indicated by similar presence of nuclear atypia (see yellow ovals) at the conclusion of the study were not observed (**Supplementary Figure 7L**). Lastly, as in other experimental models of NAFLD (**Figures 1** and **2**), Tn housing did not modify hepatic expression of insulin signaling (*Pparγ* and *Irs1*) or lipid metabolism-associated genes (*Lxrα*, *Lipe*, *Chrebp*, and *Srebp*) (**Supplementary Figures 7M, N**).

## Discussion

In this comparative study, we built upon our previous findings that demonstrated the ability of Tn housing (which allows for physiological responses to inflammatory stimuli) to modify key inflammatory responses associated with HFD driven NAFLD development and progression. Here we add to that knowledge by showing that Tn housing also modifies liver disease hallmarks in multiple experimental mouse models of NAFLD.

HFD feeding is favored in experimental models of NAFLD as it induces robust obesity, obesity-associated metabolic sequelae, and hepatic steatosis. However, a major shortcoming of this model is that even prolonged feeding does not induce severe steatohepatitis or hepatic fibrosis (40, 44). NASH diet is composed of high fat content coupled to high content of carbohydrates – a dietary composition that more accurately mimics human dietary intakes and is believed to more closely recapitulate NAFLD development progression. However, an apparent caveat of this model is that the higher cholesterol content is overwhelming and does not represent physiological levels found in human diets (45). Overnutrition, commonly seen in diet induced obesity models, significantly alters cellular metabolism (46). Altered cellular metabolism profoundly shapes immune cell function (24, 46). Specifically, in the context of NAFLD, obesity drives ectopic fat deposition into the liver and subsequent immune cell infiltration (47, 48). Our findings demonstrate that Tn housing was sufficient to augment obesity, ALT, hepatic immune cell accrual and the overall liver tissue damage in during NASH diet feeding. Specifically, we demonstrate that Tn housing was sufficient to not only promote increased obesity over time but to also increase hepatic accrual of macrophages and neutrophils which correlates with elevated ALT and the overall NAS severity. However, the signaling mechanisms shaping the altered NAFLD kinetics and severity under Tn housing conditions that uniquely promote increased hepatic myeloid cell accrual remain unknown. During NASH/NAFLD, liver resident macrophages (Kupffer cells) decrease and are replaced by recruited circulating monocyte-derived macrophages. These macrophages are distinct from Kupffer cells, and their presence is associated with worsened liver disease (49). If and how Tn housing modifies the recruitment of these distinct macrophage populations and their functional capabilities is unknown. In addition, investigation of the impact of Tn housing on environmental cues or other non-immune cell factors which may contribute to worsened disease pathogenesis is warranted. Tn housing was not sufficient to induce hepatic fibrosis under NASH diet feeding conditions. Hence, future studies should focus on the role of Tn housing on activation of other liver resident cells known to contribute to fibrosis development including stellate cells (50). Additionally, the contribution of other immune cells to hepatic inflammation and fibrosis outside the scope of this study should be investigated (51–54).

Although MCD diet induces robust hepatic steatosis, inflammation, and fibrosis, a major disadvantage of this model is that it induces chronic weight loss and lacks the presence of metabolic syndrome – parameters frequently associated with obesity-driven liver disease in humans. However, given the increase in cases of non-obese individuals with NAFLD or “lean NAFLD,” this model is again garnering more attention (55, 56). Notably, lean (BMI<25kg/m^2^) and non-obese (BMI<30kg/m^2^) individuals with NAFLD make up 5.1% and 12.1% of all NAFLD cases respectively in the general population (57). These individuals, in addition to liver disease, display worse type 2 diabetes, metabolic syndrome, cardiovascular disease, and fibrosis compared to their obese counterparts (58, 59). Such reports seemingly uncouple obesity from NAFLD and validate the use of MCD as a tool to understand disease progression in “lean NAFLD”. We show that Tn housing in context of MCD diet was sufficient to amplify hepatic steatosis, hepatocellular ballooning, lobular inflammation, overall NAS severity, and hepatic fibrosis. Moreover, these effects were amplified under Tn conditions after prolonged exposure to MCD diet feeding. However, we did not observe differences in fibrosis during prolonged MCD diet exposure. Given that observed reduction in macrophage inflammation under these conditions, prolonged exposure to MCD-driven NAFLD milieu could contribute to immune cell exhaustion and diminished hepatic fibrosis (60). Notably, combined deficiency of choline and methionine impairs β-oxidation and decreases secretion of VLDL contributing to increased fatty liver, cytokine secretion, inflammation, and development of fibrosis (9). Additionally, β−oxidation regulates immune cell inflammation and function (26, 61, 62). If and how Tn housing regulates β-oxidation, subsequent immune responses and VLDL secretion remains unknown.

Chemicals such as CCl_4_ are effective triggers of hepatic fibrosis and have been heavily utilized to study liver disease progression. Cellular toxicity, a major caveat of this method, is known to induce generation of reactive metabolites, ROS, oxidative stress and imbalances in cellular damage and regeneration. These processes can subsequently activate hepatic stellate cell proliferation and induce development and progression of hepatic fibrosis (42, 63). We show that both Tn and Ts housing in the context of high dose CCl_4_ administration drive similar hepatocellular necrosis and fibrosis. Despite the histopathological similarities, Tn housing blunted systemic ALT level. ALT, abundantly found in the cytosol of hepatocytes, is released upon hepatocellular damage and has a half-life of approximately 47 hours in the blood, with levels varying 10-30% within a given day (64). Whether the ALT levels are attributed to altered cycles of hepatocellular regeneration under Ts and Tn housing conditions remains unknown. Housing temperature also regulates inflammatory signaling cascades (e.g., NF-κB, TNFα IL-6) that drive the initial priming necessary to stimulate hepatocyte regeneration (65). As such, the contribution of Tn housing to hepatocellular damage and regeneration in the context of CCl_4_ treatment warrants further investigation. Additionally, although ALT is often recognized as reliable marker of liver disease, ALT levels do not always directly correlate with disease progression. In the context of hepatitis B and C infection, some individuals display normal ALT levels even with the presence of advanced fibrosis (66, 67). As such, despite the invasive nature of the procedure, liver biopsy remains the gold standard in determining NAFLD diagnosis and disease severity.

Recently, low dose CCl4 in combination with WD feeding (containing cholesterol) is utilized to induce hepatic fibrosis and mimic human NASH in C57BL/6 wild type mice – a process uncharacteristic of WD feeding alone (5). Diet induced obesity is a known modifier of hepatic immune cell accrual including macrophages, which is further exacerbated under Tn housing conditions. Interestingly, in the context of Tn housing our data shows a reduction in total hepatic macrophage accrual in WD+CCl_4_ treated mice. CCl_4_ treatment modifies immune cell function (43, 68). How Tn housing modulates hepatic immune cell function during WD+CCl_4_ combination remains to be studied. Although no differences in histological fibrosis were observed, increased expression of fibrosis, cell proliferation and HCC-associated genes under Tn conditions was noted. These data suggest that prolonged exposure may be needed to fully uncover Tn housing mediated WD+CCl_4_ treatment effects on fibrosis. Overall, our findings with WD+CCl_4_ agree with published reports (5), apart from serum ALT analysis. We show that WD+CCl_4_ elevates serum ALT in Ts conditions over the control group (WD+Oil). This could be due in part to differences in host gut microbiome, time of day of serum collection or hepatocellular cycles of damage and regeneration (69–71). In the context of Tn housing, we demonstrate that WD+CCl_4_ treatment blunts ALT levels. These data suggest that under Tn conditions the WD feeding may contribute to activation of other mechanisms subsequently offering some protection against the observed increased ALT seen in Ts housed counterparts. However, it should be noted that hepatic histopathological manifestations due to CCl4 use are not consistent with NAFLD histopathology seen in humans. Hence, these key limitations should be accounted for when using CCl_4_ to model NAFLD associated histopathology.

The molecular mechanisms that contribute to NAFLD progression are still under investigation. Steatosis development is due in part to both extra- and intrahepatic processes. Hormone sensitive lipase (HSL) regulates adipose tissue TG hydrolysis into free fatty acids (FFAs) which are subsequently taken up by the liver (72, 73). Insulin inhibits HSL activity during feeding, hence during the low insulin fasting state HSL activity is increased and facilitates fatty acid release (72). Indeed, inhibition of HSL in mice decreases plasma FFA concentration and reduces hepatic steatosis (72, 74). Additionally, rest (i.e., non-exercise conditions) prompts fatty acid (FA) release and creates an imbalance of FAs in circulation versus their rate of oxidation. In our studies no significant changes in hepatic HSL (*Lipe*) or insulin signaling activity (*Irs1 and Pparγ*) between Ts and Tn housed groups were observed. These data suggests that Tn housing does not dominantly impact TG hydrolysis into FFA and subsequent uptake by the liver contributing to hepatic steatosis. It may be that lipoprotein lipase (LPL), which regulates intravascular hydrolysis of plasma chylomicron and very low-density lipoprotein (VLDL) into FAs for subsequent uptake by the adipose tissue and the liver, may be modified by Tn housing (72). In fact, in experimental models of atherosclerosis Tn housing has been shown to alter low-density lipoprotein composition (15). However, given limited changes in expression of insulin signaling genes between Ts and Tn housed groups in our models (something that promotes LPL activity (72), such studies require further investigation. Intrahepatic perturbation in lipid metabolic processes can also regulate hepatic steatosis. High sucrose feeding has been shown to promote steatosis *via de novo* lipogenesis. Moreover, inhibition of glucose-6-phosphatase causes hepatic entrapment of glucose and subsequently promotes *de novo* lipogenesis and hepatic steatosis. Indeed, carbohydrate-responsive element-binding protein (*Chrebp*) is activated by glucose and promotes *de novo* lipogenesis (75). Yet, our data does not uncover differences in *Chrebp* between Ts and Tn housed groups. Analysis of other genes known to regulate lipid lipogenesis including sterol regulatory element binding protein and liver x receptors (*Srebp* and *Lxrα*) (76, 77) similarly did not reveal difference in expression between Ts and Tn housed groups. Collectively, these studies may suggest that Tn housing possibly regulates development of hepatic steatosis *via* novel mechanisms. Of note, dietary insults may regulate DNA methylation which heavily relies on S-adenosylmethionine (SAM) availability and methyl donors from foods. Deficiency in folate, one of such methyl donors, is known to contribute to hepatic triglyceride accumulation *via* regulation of fatty acid synthesis genes (78, 79). Thus, whether Tn housing modifies diet associated regulation of epigenetic mechanisms contributing to NAFLD is underdefined.

Housing temperature exerts profound effects in shaping immune responsiveness. Cold stress is associated with norepinephrine release, activation of beta-adrenergic receptors on immune cells and subsequent regulation of immune cell function (80). In the context of HFD driven NAFLD, Tn housing significantly lowered expression of genes central to glucocorticoid receptor (GR) and beta 3 adrenergic receptor signaling. Moreover, Tn housing resulted in lower serum concentrations of the immunosuppressive glucocorticoid corticosterone and expression of genes that inhibit inflammation (*GR and beta 2 adrenergic receptor (β2AR*) in the spleen (3). Notably, immune cells deficient in GR or β2AR exhibit exacerbated inflammatory cytokine production following LPS stimulation (81, 82). Additionally, sequencing of peripheral blood mononuclear cells from Ts and Tn housed mice showed that Tn housing promoted increased expression of genes that negatively regulate immune responses (3). Whether the same mechanisms are conserved in other experimental models of NAFLD remains underdefined and should be examined.

Temperature modifies epigenetic regulation of immune response. It has been reported that optimally (25°C) challenged fish displayed increased IgM^+^ B cell secretion, macrophage inflammation and recovery following viral infection compared to sub-optimally (17°C) challenged fish. Notably, fish that survived infection during a suboptimal challenge exhibited significantly increased H3 and H4 histone modifications compared to that of optimally challenged fish. Specifically, they found that suboptimal challenge resulted in H3K9ac displaying transcriptional competency, activation of trained immunity H3K4me3, and enrichment of H3 histone-lysine 4 mono-methylation (H3K4me1), and a robust re-stimulatory immune response. Essentially, all assayed H4 modifications were significantly higher in sub-optimally challenged infected fish compared to optimally challenged infected fish. Moreover, these fish had more methylation along cytosine residues compared to optimally challenged fish, suggesting the role of epigenetics and subsequent activation of trained immunity in convalescing sub optimally challenged fish (83). Thus, temperature may act as a potent regulator of epigenetic mechanisms contributing to regulation of immune responses during viral infection. Whether Tn housing modifies epigenetic activity in murine immune cells and their contribution to NAFLD pathogenesis represents an attractive area of investigation.

Febrile response to infection is preserved in mammals with evidence invoking existence of beneficial response to infection (84). In fact, the available data suggests that an elevated temperature (37.5°C to 39.4°C) in ICU patients is associated with better outcomes to infectious insult compared to normothermia or hyperthermia (above 40°C) (84–86). In elderly, increased pneumonia related mortality is observed in those who lacked fever (29%) compared to those who developed a febrile response (4%). Thus, tese data suggests that internal temperatures may indeed impact the immune response or slow pathogen virulence. However, there is insufficient data to determine the impact of environmental temperature on modulation of immune responses and development of disease, specifically NAFLD, under steady state conditions in humans. Importantly, divergence in dietary consumption and lifestyle among various populations, given its relevance to NAFLD development, represents a key obstacle in addressing the impact of temperature on NAFLD severity in humans. Hence, additional epidemiological studies are required across various geographical landscapes to begin to draw correlations between NAFLD prevalence and climate.

In summary, our data demonstrates that Tn housing modifies hepatic immune cell accrual, lobular inflammation, fibrosis and overall amplifies liver tissue damage severity in experimental models of NAFLD in mice. Specifically, we demonstrate that Tn housing modifies hepatic immune cell inflammation across all models studied. Further investigation is required to complete in depth characterization of immune cell subsets and their genomic and transcriptional landscapes. These data are relevant for future comparisons of immune response in experimental models with human disease. Thus, the utility of thermoneutral housing as a means of modeling physiological murine immune responses to various inflammatory stimuli is becoming more recognized. Together, the initial findings of our studies when coupled to future investigation on this topic might serve as a foundation for interrogating how Tn housing instructs immune cell function in the context of liver inflammation and disease in multiple settings. Collectively, these insights may allow for the development of future therapies to NAFLD *via* improved understandings of immune mechanisms underlying disease development and progression.

## Data availability statement

The original contributions presented in the study are included in the article/**Supplementary Material**. Further inquiries can be directed to the corresponding author.

## Ethics statement

The animal study was reviewed and approved by Cincinnati Children’s Hospital Medical Center IACUC.

## Author contributions

JO, MM-F, DG and SD contributed to conception and design of the study. JO, KS, DG, PA, MD, MM-F, and TS executed the experimental plan and contributed to subsequent data analyses. JO, KS, and DG performed the data and statistical analyses. SS performed histological scoring and analyses. JO, KS, MM-F and SD wrote the manuscript. All authors contributed to the article and approved the submitted version.

## Supplementary material

Supplementary Figure 1 Tn housing does not impact liver damage in chow mice. Eight-week-old WT mice were fed a chow diet and housed at Ts or Tn for 24 weeks. During the course of the 24 weeks, **(A)** body weights were recorded. At the conclusion of the study, **(B)** wet liver weights were recorded, and **(C)** serum ALT levels were measured. In bar graphs, data represent mean + SEM. Representative of 3 individual experiments (n=8/condition). **(A–C)** Student’s t-test. **P<0.001.

Supplementary Figure 2 Related to Main Figure 1: Characterization of liver disease in Ts and Tn housed NASH diet fed mice. At the conclusion of the study, additional parameters of NAFLD severity were analyzed. **(A)** Wet weights of epididymal, inguinal and perirenal white adipose tissues, and **(B)** wet liver weights. **(C)** Hepatic triglycerides levels measured in homogenized tissue lysates. **(D–H)** Hepatic inflammation as defined by gene expression and hepatic immune cell accrual and inflammatory cytokine production at the conclusion of the study. qPCR analysis of liver **(D)** *Tnfa* and **(E)** *Ccl2* and *Ccl3* expression. **(F)** Absolute numbers of hepatic immune cells quantified *via* hemacytometer. **(G, H)** Hepatic immune cells were stimulated for 4 hours with Phorbol 12-myristate 13-acetate (PMA; 50 ng/ml) and Ionomycin (1 μg/ml), in presence of Brefeldin A (10 μg/mL) and analyzed for cytokine production by flow cytometry. **(G)** Frequency of F4/80^+^CD11b^hi^TNFa^+^ expressing cells and **(H)** frequency of CD4^+^TNFa^+^, CD4^+^IFNg, and CD4^+^IL-17a^+^ cells. **(I, J)** Quantification of hepatic fibrosis. qPCR analysis of liver **(I)** *Col1a1*, *Col1a2*, and *Acta2* expression. **(J)** (Top and Bottom) Steatosis on trichrome stain, with no pericentral or periportal fibrosis, inflammation or cholangiolar proliferations. Top, black bar = 39µm. Bottom, black bar = 42µm. **(K, L)** Quantification of insulin signaling and lipid metabolism-associated genes in the liver. qPCR analysis of liver (K) *Pparg* and *Irs1*, **(L)** *Chrebp*, *Srebp*, *Lxra* and *Lipe* expression. In bar graphs, data represent mean +/- SEM. Representative of 2 individual experiments (n=4-6/condition). **(A)** and **(C–E)** and **(G–I)** One-way ANOVA. *P<0.05, **P<0.01 ***P<0.001, ****P<0.0001. **(B, F)** and **(I, K-L)** Student’s t-test. *P<0.05, **P<0.01, ***P<0.001, ****P<0.0001

Supplementary Figure 3 Related to Main Figure 1: A representative plots of flow cytometry gating strategy of hepatic immune cells. Liver infiltrated immune cells were isolated as described in the materials and methods section. Single cell suspensions were stained with viability dye and surface markers antibody cocktail for 30 minutes at 4°C. Cells were then washed in PBS + 2% FBS and then prepared for reading on cytometer. A minimum of 200,000 cells were recorded in each tube.

Supplementary Figure 4 Related to Main Figure 2: Characterization of liver disease in Ts and Tn housed mice on short-term MCD diet. At the conclusion of the study, additional parameters of NAFLD severity were analyzed. **(A)** Liver to body weight ratio was quantified and **(B)** hepatic triglyceride levels were measured in homogenized tissue lysates. **(C)** Hepatocellular damage as quantified by serum AST levels collected at the conclusion of the study. **(D–H)** Hepatic inflammation as defined by gene expression and hepatic immune cell accrual and inflammatory cytokine production at the conclusion of the study. qPCR analysis of liver **(D)** *Tnfa* and **(E)** *Ccl2* and *Ccl3* expression. **(F)** Absolute number of hepatic immune cells were quantified using a hemacytometer. **(G, H)** Hepatic immune cells were stimulated for 4 hours with Phorbol 12-myristate 13-acetate (PMA; 50 ng/ml) and Ionomycin (1 μg/ml), in presence of Brefeldin A (10 μg/mL) and analyzed for cytokine production by flow cytometry. **(G)** Frequency of F4/80^+^CD11b^hi^TNFa^+^ expressing cells and **(H)** frequency of CD4^+^TNFa^+^, CD4^+^IFNg, and CD4^+^IL-17a^+^ cells. **(I)** Quantification of hepatic fibrosis. qPCR analysis of liver *Col1a1*, *Col1a2*, and *Acta2* expression. **(J, K)** Quantification of insulin signaling and lipid metabolism-associated genes in the liver. qPCR analysis of liver **(J)** *Pparg* and *Irs1*, and **(K)** *Chrebp*, *Srebp*, *Lxra* and *Lipe* expression. In bar graphs, data represent mean +/- SEM. (A-K) Data combined from 2 individual experiments, (n=8/condition). **(A–D)** and **(F–H)** One-way ANOVA. *P<0.05, **P<0.01 ***P<0.001, ****P<0.0001. **(I–K)** Student’s t-test. *P<0.05, **P<0.01, ***P<0.001.

Supplementary Figure 5 Related to Main Figure 2: Characterization of Ts and Tn housed mice on prolonged MCD diet. **(A)** Schematic of experimental design. Eight-week-old WT mice maintained at Ts or acclimated to Tn for 2 weeks and fed a chow (baseline reference) or MCD diet for 10 weeks. During the course of the 10 weeks **(B)** body weight was recorded. At the conclusion of the study, additional parameters of NAFLD severity were analyzed. **(C)** Liver to body weight ratio was recorded, **(D)** hepatic triglyceride levels were measured in homogenized tissue lysates and **(E)** hepatocellular damage was quantified by serum ALT and **(F)** AST levels. **(G)** The liver tissue was preserved in formalin, stained with hematoxylin eosin (H&E), and analyzed by a clinical pathologist. Table depicting histological scoring analyses for fibrosis, lobular inflammation, hepatocellular ballooning, macrovesicular (MV) steatosis grade, steatosis percentage, portal inflammation and NAFLD activity score (NAS) severity. **(H–M)** Hepatic inflammation as defined by gene expression and hepatic immune cell accrual and inflammatory cytokine production at the conclusion of the study. qPCR analysis of liver **(H)** *Tnfa* and **(I)** *Ccl2* and *Ccl3* expression. **(J)** Absolute number of hepatic immune cells were calculated using a hemacytometer. **(K–M)** Hepatic inflammation as defined by flow cytometric analyses of hepatic immune cell accrual. **(K)** Donut charts depicting liver immune compositions (macrophages, neutrophils, CD4^+^ cells, CD8^+^ cells, and unidentified cells) as determined by flow cytometry. Cells were stimulated for 4 hours with Phorbol 12-myristate 13-acetate (PMA; 50 ng/ml) and Ionomycin (1 μg/ml), in presence of Brefeldin A (10 μg/mL) and analyzed for cytokine production. **(L)** Hepatic frequency of F4/80^+^CD11b^hi^TNFa^+^ expressing cells and **(M)** hepatic frequency of CD4^+^TNFa^+^, CD4^+^IFNg, and CD4^+^IL-17a^+^ cells. **(N)** Quantification of hepatic fibrosis by qPCR analysis using C*ol1a1*, *Col1a2* and *Acta2* expression. In bar graphs, data represent mean +/- SEM. For box plots, the midline represents the mean, boxes represent the interquartile range. **(B–N)** Data combined from 2 independent experiments (n=6-8/condition). **(C, D)** and **(F–H)** and **(J–M)** One-way ANOVA. *P<0.05, **P<0.01 ***P<0.001. One-way ANOVA. *P<0.05, **P<0.01. **(I, N)** Student’s t-test. *P<0.05, **P<0.01.

Supplementary Figure 6 Tn housing decreases hepatocellular ALT release in CCl_4_ model of liver injury. **(A)** Schematic of experimental design. Eight-week-old WT mice maintained at Ts or acclimated to Tn for 2 weeks and treated with either olive oil (vehicle control) or CCL_4_ i.p. 2x weekly for 3 weeks. **(B)** Terminal body weight recorded at the conclusion of the study. **(C, D)** The liver tissue was preserved in formalin, stained with hematoxylin eosin (H&E), and analyzed by a clinical pathologist. **(C)** Table depicting histological scoring analyses for fibrosis, lobular inflammation, hepatocellular ballooning, macrovesicular (MV) steatosis grade, steatosis percentage, portal inflammation and NAFLD activity score (NAS) severity. **(D)** Liver H&E staining. (Top) Immune cell infiltrate. Black bar = 82µm. (Bottom) Hepatocellular necrosis as indicated by acidophil bodies (yellow arrows). Black bar = 52µm. **(E)** Hepatocellular damage as quantified by serum ALT levels collected at the conclusion of the study. **(F–K)** Hepatic inflammation as defined by gene expression and hepatic immune cell accrual and inflammatory cytokine production at the conclusion of the study. qPCR analysis of liver **(F)** *Tnfa* and **(G)** *Ccl2* and *Ccl3* expression. **(H)** Absolute number of hepatic immune cells were calculated using a hemacytometer. **(I)** Donut charts depicting liver immune compositions (macrophages, neutrophils, CD4s, CD8s, NKs, NKTs, B cells, and unidentified cells) as determined by flow cytometry from percent of CD45^+^ population. **(J, K)** Cells were stimulated for 4 hours with Phorbol 12-myristate 13-acetate (PMA; 50 ng/ml) and Ionomycin (1 μg/ml), in presence of Brefeldin A (10 μg/mL) and analyzed for cytokine production by flow cytometry. **(J)** Hepatic frequency of F4/80^+^CD11b^hi^TNFa^+^ and **(K)** hepatic frequency of CD4^+^TNFa^+^, CD4^+^IFNg, and CD4^+^IL-17a^+^ cells. **(L, M)** Quantification of hepatic fibrosis. qPCR analysis of liver **(L)** *Col1a1*, *Col1a2* and *Acta2* expression. **(M)** (Top) Trichrome staining. Zone 3, hepatocellular necrosis with inflammation and fibrosis. Black bar = 256µm. (Bottom) On left side, hepatocellular necrosis with pericellular fibrosis and inflammation, imparting a stellate outline; scattered foamy macrophages. On right side, hepatocytes preserved in zone 1. Black bar = 35µm. In bar graphs, data represent mean +/- SEM. **(B, E–L)** Data combined from 2 individual experiments (n=7-8/condition). **(C-, D, M)** Data combined from 2 individual experiments (n=3-4/condition). **(E, F, H)** and **(J, K)** One-way ANOVA. **P<0.01. **(G, L)** Student’s t-test. *P<0.05, **P<0.01.

Supplementary Figure 7 Related to Main Figure 3: Characterization of liver disease in Ts and Tn housed mice during WD+CCl_4_ treatment. At the conclusion of the study, additional parameters of NAFLD severity were analyzed. **(A)** Wet liver weights were recorded at the conclusion of the study and **(B)** hepatic triglyceride levels were measured in homogenized tissue lysates. **(C)** AST levels were measured in the serum. **(D–H)** Hepatic inflammation as defined by gene expression and hepatic immune cell accrual and inflammatory cytokine production at the conclusion of the study. qPCR analysis of liver **(D)** *Tnfa* and **(E)** *Ccl2* and *Ccl3* expression. **(F)** Absolute number of hepatic immune cells was calculated using a hemacytometer. **(G, H)** Hepatic inflammation as defined by flow cytometric analyses of hepatic immune cell accrual. Isolated hepatic immune cells were stimulated for 4 hours with Phorbol 12-myristate 13-acetate (PMA; 50 ng/ml) and Ionomycin (1 μg/ml), in presence of Brefeldin A (10 μg/mL) and analyzed for cytokine production. **(G)** Hepatic frequency of F4/80^+^CD11b^hi^TNFa^+^ expressing cells and **(H)** hepatic frequency of CD4^+^TNFa^+^, CD4^+^IFNg, and CD4^+^IL-17a^+^ cells. **(I)** Quantification of hepatic fibrosis using qPCR analysis of liver *Col1a1*, *Col1a2* and *Acta2* expression. **(J)** Quantification of hepatocellular regeneration using qPCR analysis of liver *Ki67* and *Ccnd1* expression. **(K)** Quantification of hepatocellular carcinoma genes using qPCR analysis of liver *H19* and *Afp* expression. **(L)** Liver hematoxylin and eosin (H&E) staining. (Top) Bile ductular (cholangiolar) proliferations with associated pericellular delicate fibrosis apparent in the right side of the field. Black bar = 332µm. (Bottom) Nuclear inclusions (including in binucleated forms) (orange arrows), nuclear atypia overlapping with patterns of steatosis (yellow ovals), in zone 3, in a background of cholangiolar proliferations. Black bar = 31µm. **(M, N)** Quantification of insulin signaling and lipid metabolism-associated genes in the liver. qPCR analysis of liver (M) *Pparg* and *Irs1* and (N) *Chrebp*, *Srebp*, *Lxra* and *Lipe* expression. In bar graphs, data represent mean +/- SEM. **(A-D)** Data combined from 2 individual experiments (n=10/condition) **(E, I–J)** Data combined from 2 individual experiments (n=7-8/condition). **(F)** Data combined from 2 individual experiments (n=10-14/condition) **(G, H)** Data combined from 2 individual experiments (n=6/condition). **(M, N)** Data combined from 2 individual experiments (n=10-11/condition). **(A–H)** One-way ANOVA. *P<0.05, **P<0.005, ***P<0.001, ****P<0.0001. **(I, K)** and **(M, N)** Student’s t-test. *P<0.05, **P<0.01, ***P<0.001, ****P<0.0001.
