# Supplementary figures and images for: Thermoneutral housing shapes hepatic inflammation and damage in mouse models of non-alcoholic fatty liver disease
Source: Front Immunol. 2023 Feb 17;14:1095132. doi: 10.3389/fimmu.2023.1095132 (PMC9982161; doi:10.3389/fimmu.2023.1095132)

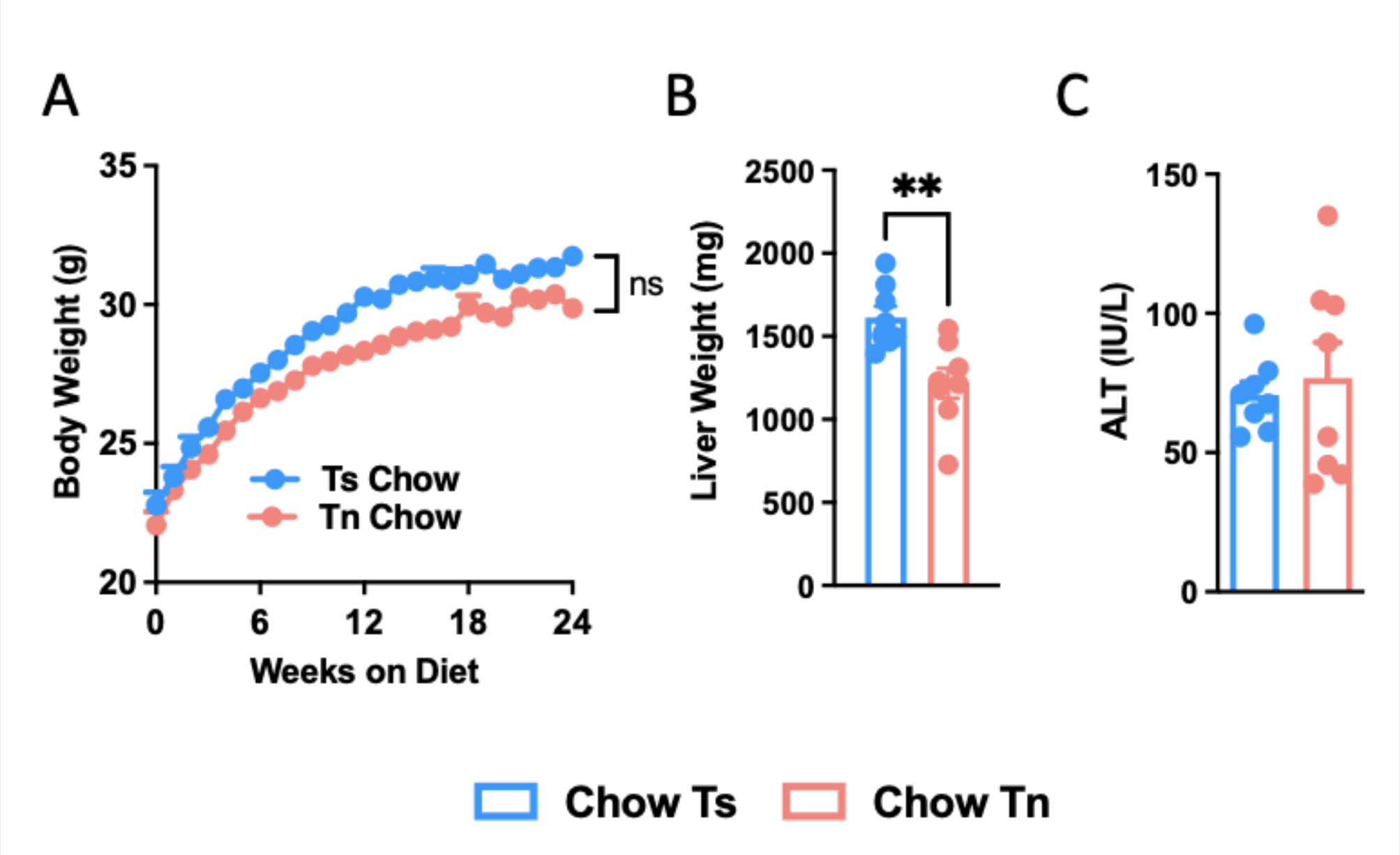

Supplement: Supplementary Figure 1 — Tn housing does not impact liver damage in chow mice. Eight-week-old WT mice were fed a chow diet and housed at Ts or Tn for 24 weeks. During the course of the 24 weeks, (A) body weights were recorded. At the conclusion of the study, (B) wet liver weights were recorded, and (C) serum ALT levels were measured. In bar graphs, data represent mean + SEM. Representative of 3 individual experiments (n=8/condition). (A–C) Student’s t-test. **P<0.001. [file Image_1.jpeg]

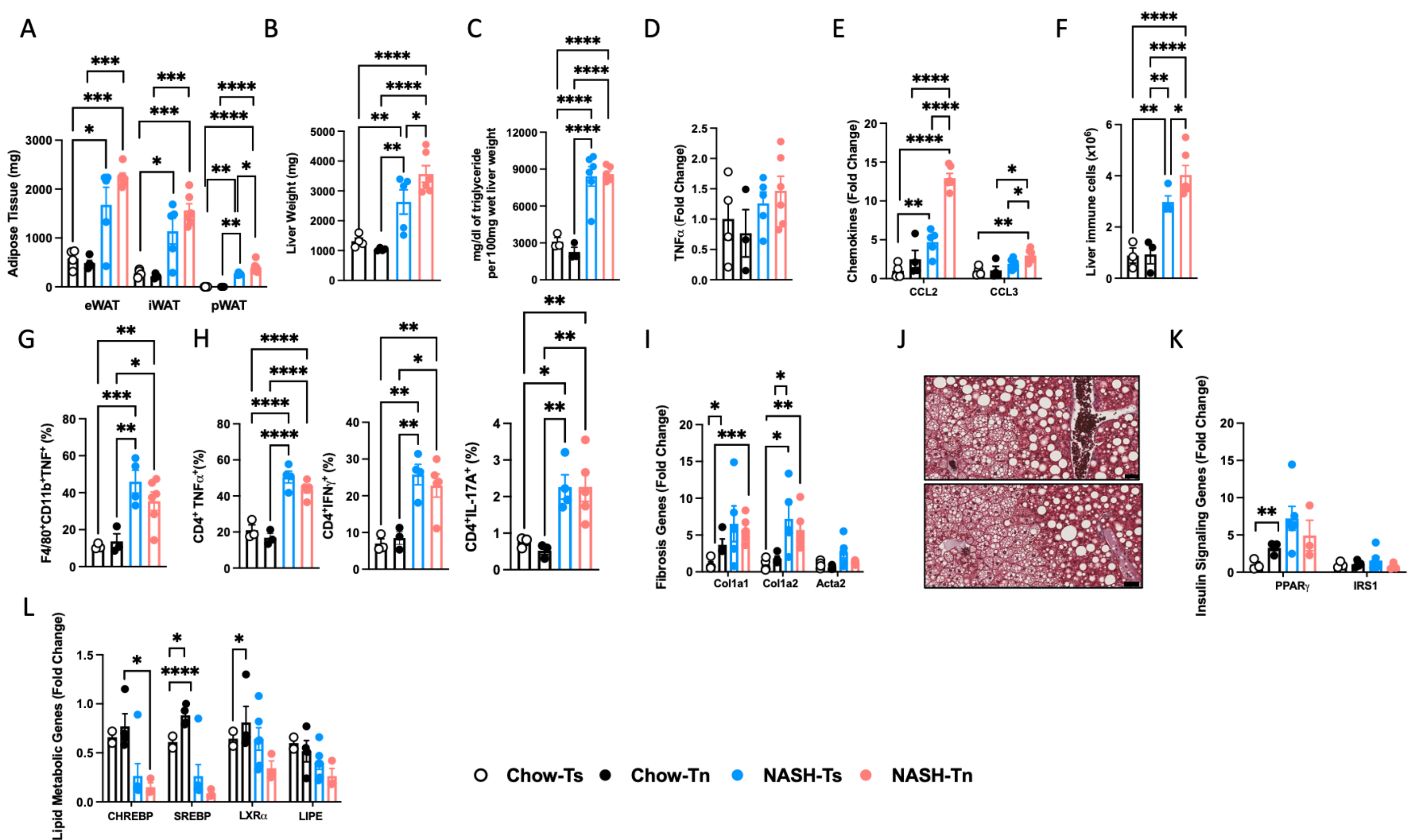

Supplement: Supplementary Figure 2 — Related to Main Figure 1: Characterization of liver disease in Ts and Tn housed NASH diet fed mice. At the conclusion of the study, additional parameters of NAFLD severity were analyzed. (A) Wet weights of epididymal, inguinal and perirenal white adipose tissues, and (B) wet liver weights. (C) Hepatic triglycerides levels measured in homogenized tissue lysates. (D–H) Hepatic inflammation as defined by gene expression and hepatic immune cell accrual and inflammatory cytokine production at the conclusion of the study. qPCR analysis of liver (D) Tnfa and (E) Ccl2 and Ccl3 expression. (F) Absolute numbers of hepatic immune cells quantified via hemacytometer. (G, H) Hepatic immune cells were stimulated for 4 hours with Phorbol 12-myristate 13-acetate (PMA; 50 ng/ml) and Ionomycin (1 μg/ml), in presence of Brefeldin A (10 μg/mL) and analyzed for cytokine production by flow cytometry. (G) Frequency of F4/80+CD11bhiTNFa+ expressing cells and (H) frequency of CD4+TNFa+, CD4+IFNg, and CD4+IL-17a+ cells. (I, J) Quantification of hepatic fibrosis. qPCR analysis of liver (I) Col1a1, Col1a2, and Acta2 expression. (J) (Top and Bottom) Steatosis on trichrome stain, with no pericentral or periportal fibrosis, inflammation or cholangiolar proliferations. Top, black bar = 39µm. Bottom, black bar = 42µm. (K, L) Quantification of insulin signaling and lipid metabolism-associated genes in the liver. qPCR analysis of liver (K) Pparg and Irs1, (L) Chrebp, Srebp, Lxra and Lipe expression. In bar graphs, data represent mean +/- SEM. Representative of 2 individual experiments (n=4-6/condition). (A) and (C–E) and (G–I) One-way ANOVA. *P<0.05, **P<0.01 ***P<0.001, ****P<0.0001. (B, F) and (I, K-L) Student’s t-test. *P<0.05, **P<0.01, ***P<0.001, ****P<0.0001 [file Image_2.jpeg]

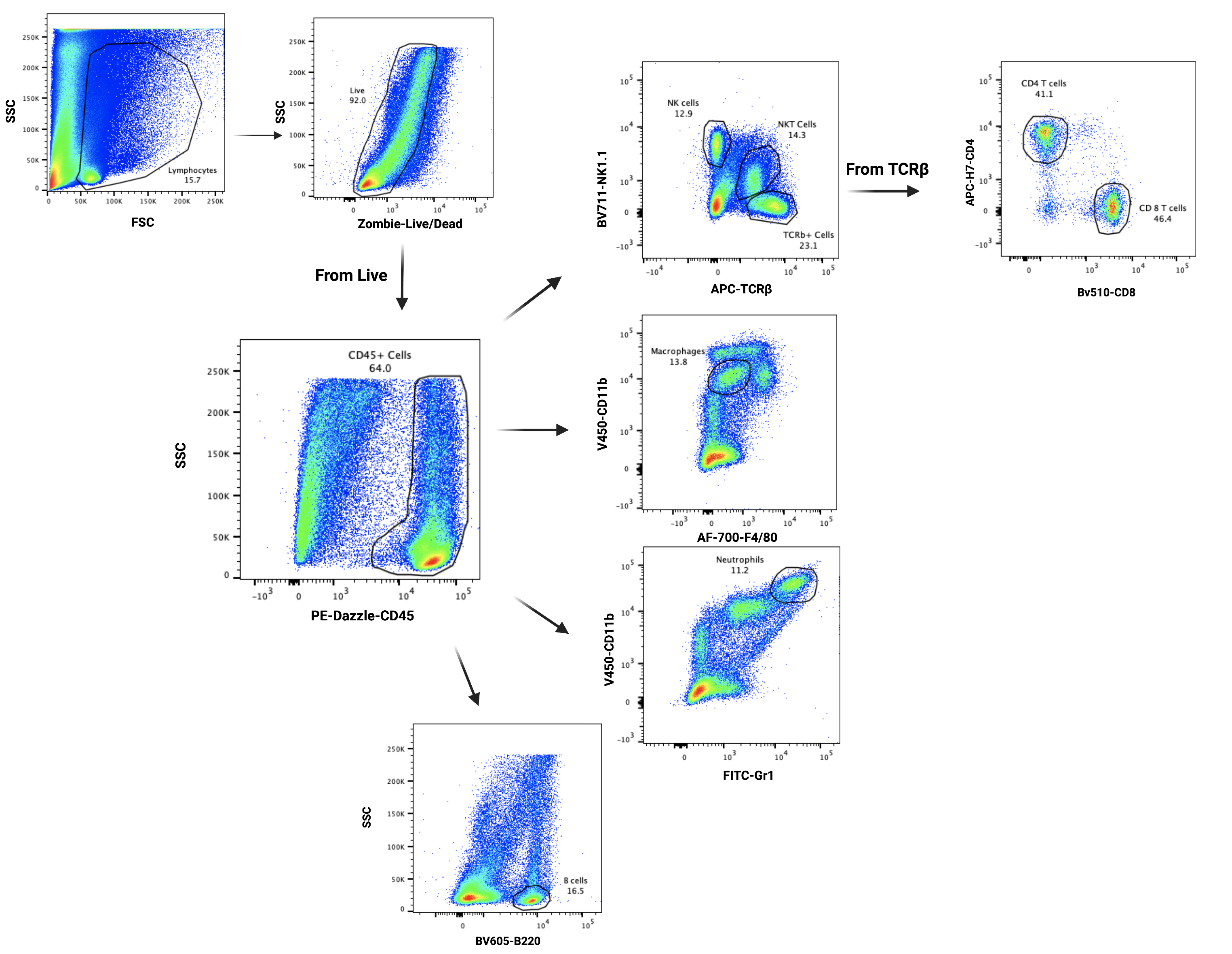

Supplement: Supplementary Figure 3 — Related to Main Figure 1: A representative plots of flow cytometry gating strategy of hepatic immune cells. Liver infiltrated immune cells were isolated as described in the materials and methods section. Single cell suspensions were stained with viability dye and surface markers antibody cocktail for 30 minutes at 4°C. Cells were then washed in PBS + 2% FBS and then prepared for reading on cytometer. A minimum of 200,000 cells were recorded in each tube. [file Image_3.jpeg]

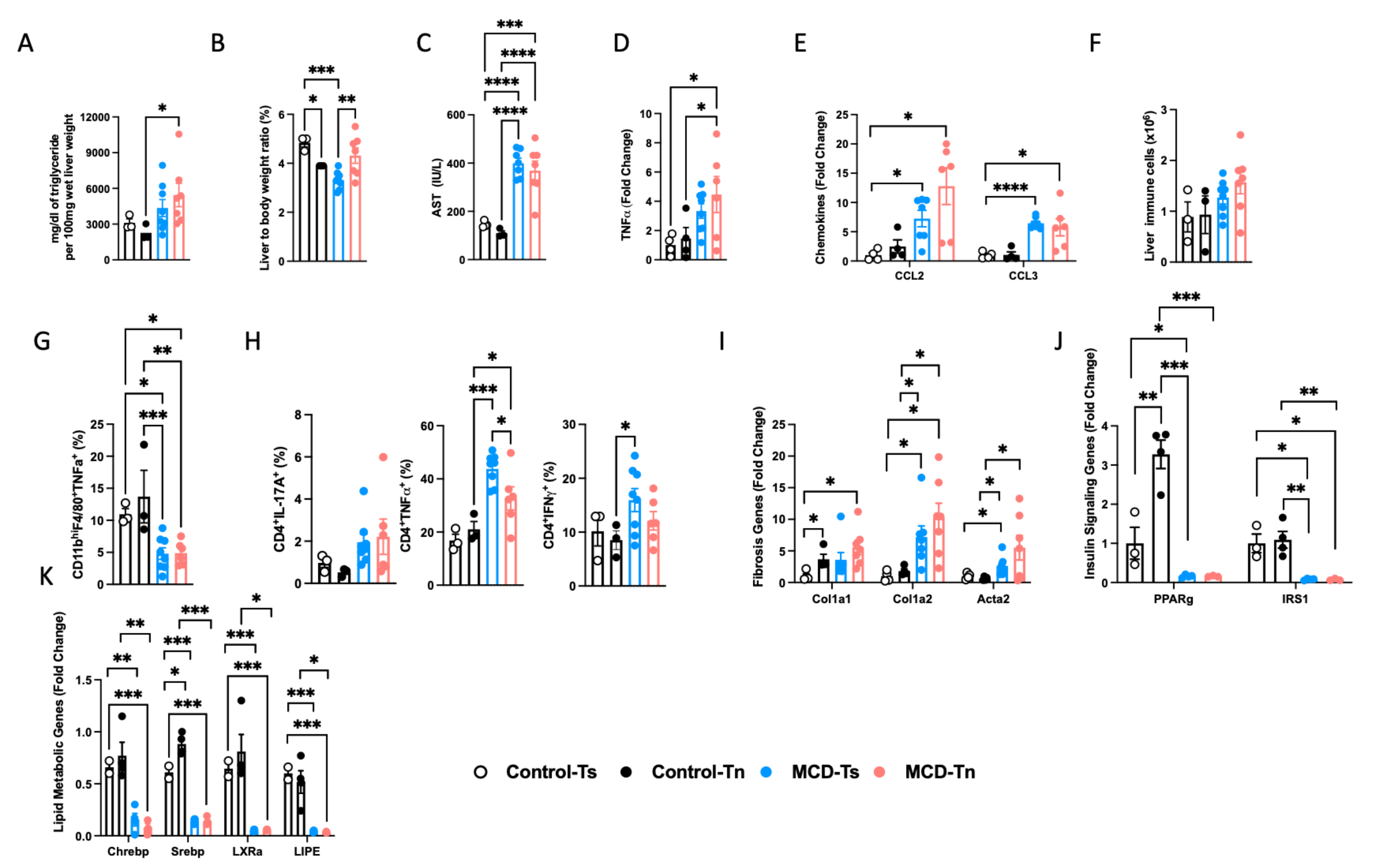

Supplement: Supplementary Figure 4 — Related to Main Figure 2: Characterization of liver disease in Ts and Tn housed mice on short-term MCD diet. At the conclusion of the study, additional parameters of NAFLD severity were analyzed. (A) Liver to body weight ratio was quantified and (B) hepatic triglyceride levels were measured in homogenized tissue lysates. (C) Hepatocellular damage as quantified by serum AST levels collected at the conclusion of the study. (D–H) Hepatic inflammation as defined by gene expression and hepatic immune cell accrual and inflammatory cytokine production at the conclusion of the study. qPCR analysis of liver (D) Tnfa and (E) Ccl2 and Ccl3 expression. (F) Absolute number of hepatic immune cells were quantified using a hemacytometer. (G, H) Hepatic immune cells were stimulated for 4 hours with Phorbol 12-myristate 13-acetate (PMA; 50 ng/ml) and Ionomycin (1 μg/ml), in presence of Brefeldin A (10 μg/mL) and analyzed for cytokine production by flow cytometry. (G) Frequency of F4/80+CD11bhiTNFa+ expressing cells and (H) frequency of CD4+TNFa+, CD4+IFNg, and CD4+IL-17a+ cells. (I) Quantification of hepatic fibrosis. qPCR analysis of liver Col1a1, Col1a2, and Acta2 expression. (J, K) Quantification of insulin signaling and lipid metabolism-associated genes in the liver. qPCR analysis of liver (J) Pparg and Irs1, and (K) Chrebp, Srebp, Lxra and Lipe expression. In bar graphs, data represent mean +/- SEM. (A-K) Data combined from 2 individual experiments, (n=8/condition). (A–D) and (F–H) One-way ANOVA. *P<0.05, **P<0.01 ***P<0.001, ****P<0.0001. (I–K) Student’s t-test. *P<0.05, **P<0.01, ***P<0.001. [file Image_4.jpeg]

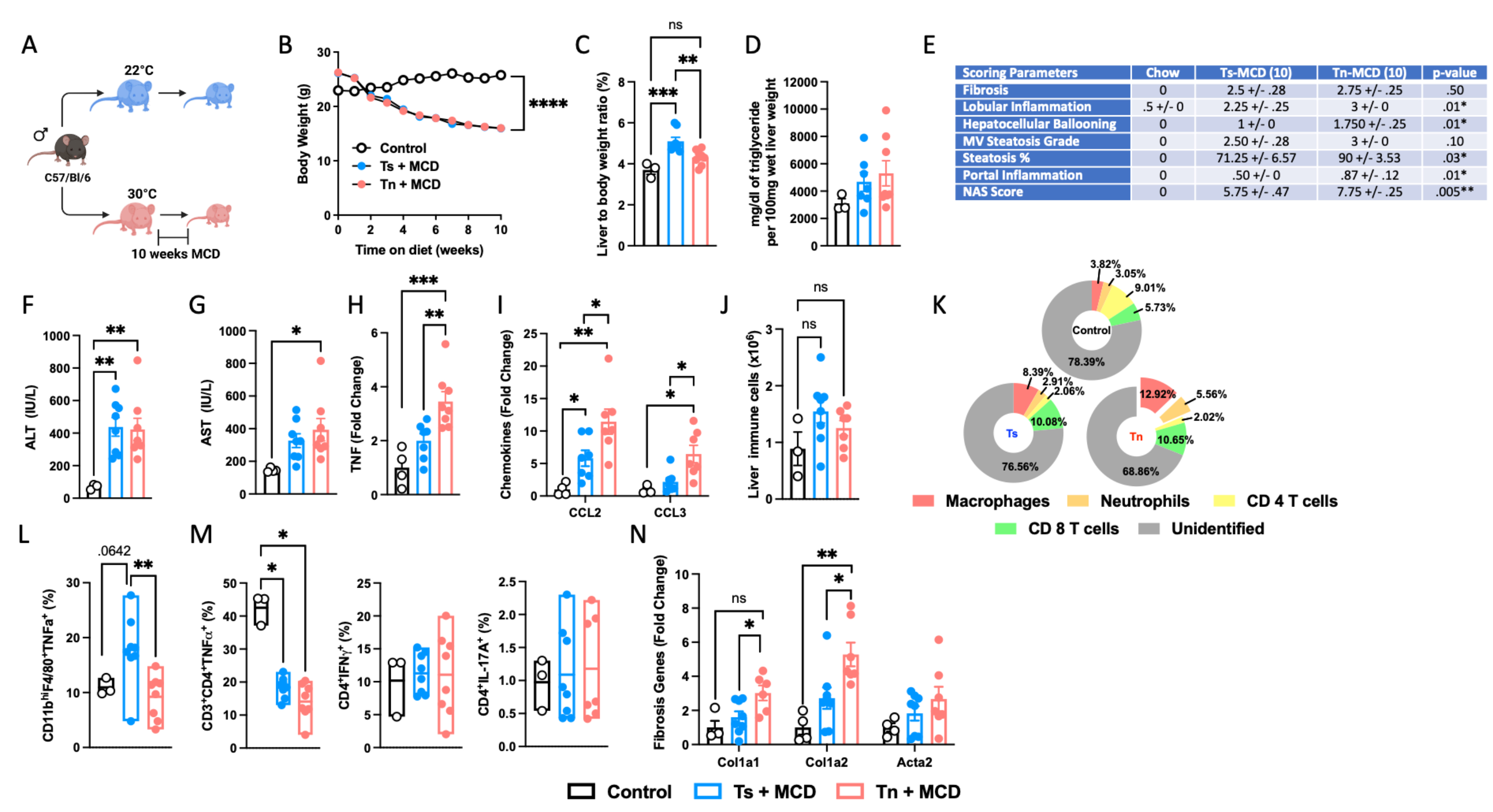

Supplement: Supplementary Figure 5 — Related to Main Figure 2: Characterization of Ts and Tn housed mice on prolonged MCD diet. (A) Schematic of experimental design. Eight-week-old WT mice maintained at Ts or acclimated to Tn for 2 weeks and fed a chow (baseline reference) or MCD diet for 10 weeks. During the course of the 10 weeks (B) body weight was recorded. At the conclusion of the study, additional parameters of NAFLD severity were analyzed. (C) Liver to body weight ratio was recorded, (D) hepatic triglyceride levels were measured in homogenized tissue lysates and (E) hepatocellular damage was quantified by serum ALT and (F) AST levels. (G) The liver tissue was preserved in formalin, stained with hematoxylin eosin (H&E), and analyzed by a clinical pathologist. Table depicting histological scoring analyses for fibrosis, lobular inflammation, hepatocellular ballooning, macrovesicular (MV) steatosis grade, steatosis percentage, portal inflammation and NAFLD activity score (NAS) severity. (H–M) Hepatic inflammation as defined by gene expression and hepatic immune cell accrual and inflammatory cytokine production at the conclusion of the study. qPCR analysis of liver (H) Tnfa and (I) Ccl2 and Ccl3 expression. (J) Absolute number of hepatic immune cells were calculated using a hemacytometer. (K–M) Hepatic inflammation as defined by flow cytometric analyses of hepatic immune cell accrual. (K) Donut charts depicting liver immune compositions (macrophages, neutrophils, CD4+ cells, CD8+ cells, and unidentified cells) as determined by flow cytometry. Cells were stimulated for 4 hours with Phorbol 12-myristate 13-acetate (PMA; 50 ng/ml) and Ionomycin (1 μg/ml), in presence of Brefeldin A (10 μg/mL) and analyzed for cytokine production. (L) Hepatic frequency of F4/80+CD11bhiTNFa+ expressing cells and (M) hepatic frequency of CD4+TNFa+, CD4+IFNg, and CD4+IL-17a+ cells. (N) Quantification of hepatic fibrosis by qPCR analysis using Col1a1, Col1a2 and Acta2 expression. In bar graphs, data represent mean +/- SEM. F [file Image_5.jpeg]

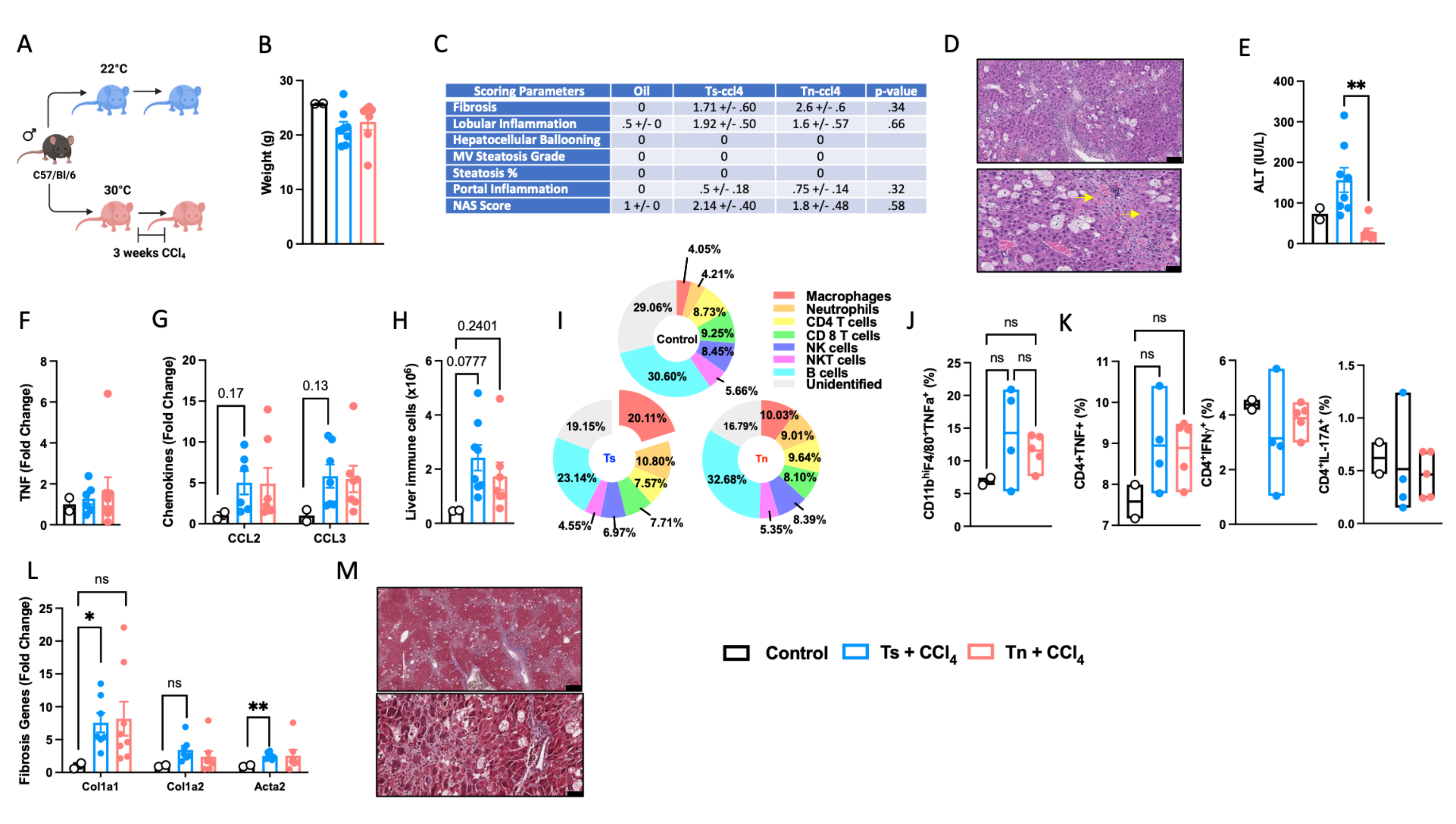

Supplement: Supplementary Figure 6 — Tn housing decreases hepatocellular ALT release in CCl4 model of liver injury. (A) Schematic of experimental design. Eight-week-old WT mice maintained at Ts or acclimated to Tn for 2 weeks and treated with either olive oil (vehicle control) or CCL4 i.p. 2x weekly for 3 weeks. (B) Terminal body weight recorded at the conclusion of the study. (C, D) The liver tissue was preserved in formalin, stained with hematoxylin eosin (H&E), and analyzed by a clinical pathologist. (C) Table depicting histological scoring analyses for fibrosis, lobular inflammation, hepatocellular ballooning, macrovesicular (MV) steatosis grade, steatosis percentage, portal inflammation and NAFLD activity score (NAS) severity. (D) Liver H&E staining. (Top) Immune cell infiltrate. Black bar = 82µm. (Bottom) Hepatocellular necrosis as indicated by acidophil bodies (yellow arrows). Black bar = 52µm. (E) Hepatocellular damage as quantified by serum ALT levels collected at the conclusion of the study. (F–K) Hepatic inflammation as defined by gene expression and hepatic immune cell accrual and inflammatory cytokine production at the conclusion of the study. qPCR analysis of liver (F) Tnfa and (G) Ccl2 and Ccl3 expression. (H) Absolute number of hepatic immune cells were calculated using a hemacytometer. (I) Donut charts depicting liver immune compositions (macrophages, neutrophils, CD4s, CD8s, NKs, NKTs, B cells, and unidentified cells) as determined by flow cytometry from percent of CD45+ population. (J, K) Cells were stimulated for 4 hours with Phorbol 12-myristate 13-acetate (PMA; 50 ng/ml) and Ionomycin (1 μg/ml), in presence of Brefeldin A (10 μg/mL) and analyzed for cytokine production by flow cytometry. (J) Hepatic frequency of F4/80+CD11bhiTNFa+ and (K) hepatic frequency of CD4+TNFa+, CD4+IFNg, and CD4+IL-17a+ cells. (L, M) Quantification of hepatic fibrosis. qPCR analysis of liver (L) Col1a1, Col1a2 and Acta2 expression. (M) (Top) Trichrome staining. Zone 3, hepatocellular necrosis with inflamm [file Image_6.jpeg]

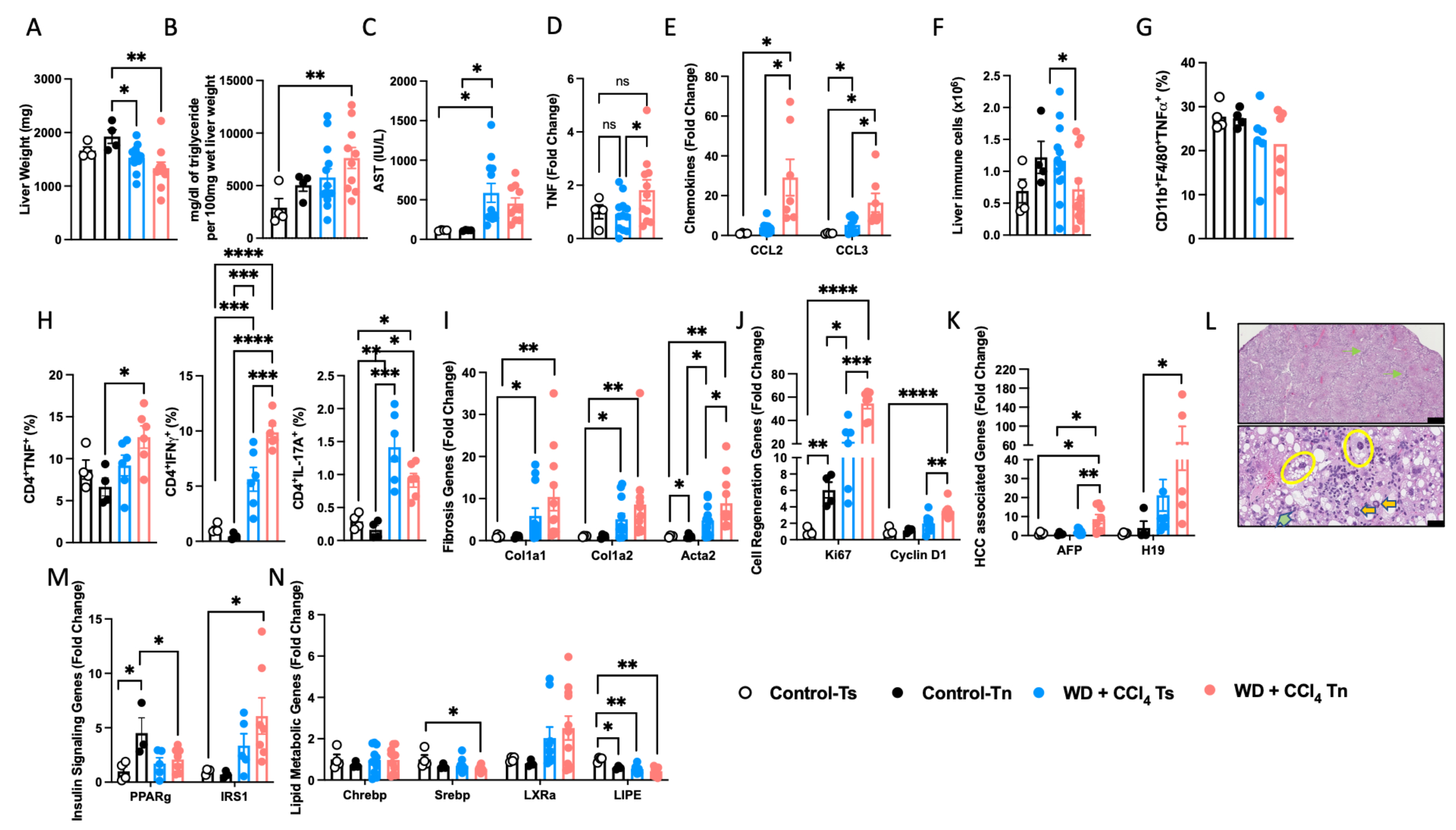

Supplement: Supplementary Figure 7 — Related to Main Figure 3: Characterization of liver disease in Ts and Tn housed mice during WD+CCl4 treatment. At the conclusion of the study, additional parameters of NAFLD severity were analyzed. (A) Wet liver weights were recorded at the conclusion of the study and (B) hepatic triglyceride levels were measured in homogenized tissue lysates. (C) AST levels were measured in the serum. (D–H) Hepatic inflammation as defined by gene expression and hepatic immune cell accrual and inflammatory cytokine production at the conclusion of the study. qPCR analysis of liver (D) Tnfa and (E) Ccl2 and Ccl3 expression. (F) Absolute number of hepatic immune cells was calculated using a hemacytometer. (G, H) Hepatic inflammation as defined by flow cytometric analyses of hepatic immune cell accrual. Isolated hepatic immune cells were stimulated for 4 hours with Phorbol 12-myristate 13-acetate (PMA; 50 ng/ml) and Ionomycin (1 μg/ml), in presence of Brefeldin A (10 μg/mL) and analyzed for cytokine production. (G) Hepatic frequency of F4/80+CD11bhiTNFa+ expressing cells and (H) hepatic frequency of CD4+TNFa+, CD4+IFNg, and CD4+IL-17a+ cells. (I) Quantification of hepatic fibrosis using qPCR analysis of liver Col1a1, Col1a2 and Acta2 expression. (J) Quantification of hepatocellular regeneration using qPCR analysis of liver Ki67 and Ccnd1 expression. (K) Quantification of hepatocellular carcinoma genes using qPCR analysis of liver H19 and Afp expression. (L) Liver hematoxylin and eosin (H&E) staining. (Top) Bile ductular (cholangiolar) proliferations with associated pericellular delicate fibrosis apparent in the right side of the field. Black bar = 332µm. (Bottom) Nuclear inclusions (including in binucleated forms) (orange arrows), nuclear atypia overlapping with patterns of steatosis (yellow ovals), in zone 3, in a background of cholangiolar proliferations. Black bar = 31µm. (M, N) Quantification of insulin signaling and lipid metabolism-associated genes in the liver. qPCR analysis of l [file Image_7.jpeg]
