# Supplementary material for: Thermoneutral housing shapes hepatic inflammation and damage in mouse models of non-alcoholic fatty liver disease
Source: Front Immunol. 2023 Feb 17;14:1095132. doi: 10.3389/fimmu.2023.1095132 (PMC9982161; doi:10.3389/fimmu.2023.1095132)
Supplement: Supplementary file 8 [file DataSheet_1.docx]

**SUPPLEMENTARY TABLES**

**Supplementary Table 1. Associated with Main Figure 1: Absolute numbers of hepatic immune cells of Ts and Tn housed mice on a NASH diet.**

| **Cell Type** | **Control** | **Ts-NASH** | **Tn-NASH** | **p-value** |
| --- | --- | --- | --- | --- |
| Macrophages | 24711 +/- 1571 | 153377 +/- 18010 | 336355 +/- 33116 | .004** |
| Neutrophils | 19763 +/- 1496 | 67030 +/- 20266 | 191125 +/- 27224 | .004** |
| CD 4 | 58358 +/- 4061 | 4955821 +/- 60035 | 431098 +/- 160051 | .46 |
| CD 8 | 36973 +/- 2195 | 624669 +/- 53731 | 660015 +/- 86250 | .004** |

**Supplementary Table 2. Associated with Main Figure 2: Absolute numbers of hepatic immune cells from Ts and Ts housed mice on MCD diet.**

| **Cell Type** | **Control** | **Ts-MCD** | **Tn-MCD** | **p-value** |
| --- | --- | --- | --- | --- |
| Macrophages | 24711 +/- 1571 | 64675 +/- 9079 | 122011 +/- 24885 | .01* |
| Neutrophils | 19763 +/- 1496 | 42444 +/- 5233 | 191125 +/- 27224 | .001** |
| CD 4 | 58358 +/- 4061 | 111544 +/- 16994 | 103421 +/- 19511 | .74 |
| CD 8 | 36973 +/- 2195 | 113547 +/- 15706 | 104844 +/- 18686 | .70 |
| NK | 43573 +/- 3609 | 151346 +/- 20240 | 152079 +/- 22796 | .97 |
| NKT | 24486 +/- 6710 | 93429 +/- 12211 | 113579 +/- 21799 | .37 |
| B cells | 205411 +/- 12658 | 243805 +/- 48747 | 273221 +/- 72421 | .71 |

**Supplementary Table 3. Associated with Main Figure 3: Absolute numbers of hepatic immune cells from Ts and Ts housed mice on WD+CCl_4_.**

| **Cell Type** | **Control** | **Ts-WD+CCl_4_** | **Tn-WD+CCl_4_** | **p-value** |
| --- | --- | --- | --- | --- |
| Macrophages | 18203 +/- 3699 | 80865 +/- 12423 | 36680 +/- 11561 | .03* |
| Neutrophils | 34787 +/- 8798 | 21224 +/- 4524 | 33866 +/- 25189 | .61 |
| CD 4 | 79289 +/- 26719 | 128363 +/- 34808 | 60933 +/- 19335 | .10 |
| CD 8 | 63732 +/- 5479 | 118677 +/- 29363 | 95115 +/- 47464 | .66 |
| NK | 67975 +/- 4631 | 173998 +/- 98432 | 44403 +/- 13329 | .19 |
| NKT | 32769 +/- 11693 | 19783 +/- 3785 | 15456 +/- 4089 | .47 |
| B cells | 376448 +/- 150304 | 150418 +/- 41208 | 130480 +/- 49317 | .62 |
